# Reviewing qualitative research approaches in the context of critical infrastructure resilience

**DOI:** 10.1007/s10669-020-09795-8

**Published:** 2021-01-24

**Authors:** R. Cantelmi, G. Di Gravio, R. Patriarca

**Affiliations:** 1 Department of Mechanical and Aerospace Engineering, Sapienza University of Rome, Via Eudossiana, 18, 00184 Rome, Italy; 2 Land Armaments Directorate, Ministry of Defence, Via di Centocelle, 301, 00187 Rome, Italy

**Keywords:** Resilience management, Resilience engineering, Critical infrastructure, Infrastructure resilience, Review

## Abstract

Modern societies are increasingly dependent on the proper functioning of critical infrastructures (CIs). CIs produce and distribute essential goods or services, as for power transmission systems, water treatment and distribution infrastructures, transportation systems, communication networks, nuclear power plants, and information technologies. Being resilient becomes a key property for CIs, which are constantly exposed to threats that can undermine safety, security, and business continuity. Nowadays, a variety of approaches exist in the context of CIs’ resilience research. This paper provides a state-of-the-art review on the approaches that have a complete qualitative dimension, or that can be used as entry points for semi-quantitative analyses. The study aims to uncover the usage of qualitative research methods through a systematic review based on PRISMA (Preferred Reporting Items for Systematic Reviews and Meta-Analyses). The paper identifies four principal dimensions of resilience referred to CIs (i.e., techno-centric, organisational, community, and urban) and discusses the related qualitative methods. Besides many studies being focused on energy and transportation systems, the literature review allows to observe that interviews and questionnaires are most frequently used to gather qualitative data, besides a high percentage of mixed-method research. The article aims to provide a synthesis of literature on qualitative methods used for resilience research in the domain of CIs, detailing lessons learned from such approaches to shed lights on best practices and identify possible future research directions.

## Introduction

Nowadays, due to the frequent occurrence of natural disasters or catastrophic events related to human activities, a global awareness on the strategic importance of Critical Infrastructures (CIs) has increasingly grown in academic and policy-making environments (Kete et al. 2018). CIs are large-scale, man-made systems that function interdependently to produce and distribute essential goods (such as energy, water, and data) and services (such as transportation, banking, and healthcare). An infrastructure is defined as critical if its incapacity or destruction has a significant impact on health, safety, security, economics, and social well-being of a state (Council Directive 2008/114/EC of 8 December 2008).

CIs are various by nature, e.g. physical-engineered, cybernetic, organisational, etc., and by environment (geographical, natural), and operational context (political/legal/institutional, economic, etc.) (Zio 2016). Examples of CIs are those providing services of energy (including generation, transmission, distribution and storage, in regard with electricity, oil and gas supply); transportation (including rail, roads, aviation and waterways); information and telecommunication (including information systems, industrial control systems (SCADA), Internet, fixed and mobile communications and broadcasting) (see e.g. Bunney et al. 2016; Hughes et al. 2016; Leu and Peter 2016; Pagán-Trinidad et al. 2019)). A failure in such an infrastructure can be even more critical because it can produce cascading failures, sending ripple effects at regional, national, or international scale. These failures are often originated by natural hazards, such as earthquakes (Kachali et al. 2012b, a) and hurricanes (Comes et al. 2013; Comes and Van De Walle 2014).

Managing CIs today means managing CIs’ resilience. Starting from its Latin etymological root on the word *resilio* (i.e. to leap back), resilience denotes the capacity of a system to recover from challenges or disruptive events. There are several definitions of resilience available in literature grounded on the concept of robustness and adaptation in line with a seminal ecology research (Holling 1973). Among them, the definition by Bruneau et al. (2003) gives a wide perspective for CIs’ management: “the ability of the system to reduce the chances of shock, to absorb a shock if it occurs and to recover quickly after a shock (re-establish normal performance)”. This ability is recognised to result from four properties (i.e. robustness, redundancy, resourcefulness, rapidity), which are inter-related through technical, organisational, and social aspects. These abilities encompass slightly different perspectives which jointly offer the opportunity to deal with micro-meso-macro level CIs’ management (Bergström and Dekker 2014): from pure technical artefacts, towards social structures made up by small groups (Zemba et al. 2019), or large organisations (Wood et al. 2019). Resilience is relevant for management because it focuses on performance levels, as well as time and cost required to reach them (Vugrin et al. 2010b). For example, within civil infrastructures, resilience is defined as “the capacity of a civil infrastructure system to minimise performance loss due to disruption, and to recover a specified performance level within acceptable predefined time and cost limits” (Gay and Sinha 2013).

Nonetheless, as for these definitions, operationalising the concept of resilience can become puzzling for CIs’ management, especially considering their interdependent nature which recalls the systems-of-systems treats. Managing resilience calls for a reconsideration of available risk management methods and models in order not to fall within the trap of reductionism and over-simplified linear modelling techniques. New frameworks are indeed needed to integrate different perspectives (e.g. topological, functional, static, dynamic) and to ensure the capacity of dealing with complexity and uncertainties (Kröger and Zio 2011). Available literature shows that such complexity is addressed by frameworks based on either qualitative or quantitative approaches, or a combination of the two. Quantitative data usually refer to historical data, design specifications, climate models, or laboratory experiments, whereas qualitative assessments come from surveyed experts or operators, i.e. community leaders, technical operators, managers, public decision makers (Cegan et al. 2017; Kurth et al. 2017). A comparison on the features of such approaches has been discussed widely. For example, Linkov and Palma-Oliveira (2017) prepared a workshop where military commanders and civilian decision makers were brought together to explore how they make resilience-driven choices on a daily and long-term basis. According to the results of the study, a qualitative approach allows for greater flexibility in applications ranging from well-known hazards to highly uncertain ones, thanks to subject matter experts’ (SMEs) judgments (Münzberg et al. 2015). These latter can overcome those frequent cases where retrieving reliable quantitative data is challenging (Häring et al. 2017). Thereafter, qualitative information might be integrated into dedicated models to define representative and synthetic indexes.

This concept can be explored more systematically via a tiered framework to resilience assessment, intended to ease policy development and favour the adoption of resilience practices (Linkov et al. 2018). This framework consists of three different tiers at which a complex problem such as CI resilience can progressively analysed. Each tier has its own specific objectives, methods, and tools. Tier I involves the use of existing data, expert judgement, and conceptual models, in order to provide a comprehensive understanding of system’s functioning. At Tier II, decision analysis methods (such as Multicriteria Decision Analysis) are utilised, e.g. the Resilience Matrix (Fox-Lent et al. 2015) or the Analytic Hierarchy Process (AHP) (Saaty 1990). Tier II encompass methods intended to reveal the structure of the system, to check scenarios, or to compare alternatives, that later on in Tier III can be further specified. The last tier seeks to provide the highest fidelity in modelling real-world systems, through, for example, system dynamics models, graph theory, Bayesian networks, or agent-based models that allow dedicated simulations. In summary, Linkov et al. (2018) is a helpful paper for classifying resilience analytics; according to this article, Tier I and Tier II can utilise both qualitative and quantitative methods, while Tier III analysis is mainly based on a quantitative approach.

Under current challenging times, where some CIs have become even more critical for our society, the study of resilience acquires a strategical role for decision-making, at any modelling tier. Research on each tier progressed widely over recent years, thanks to many scholars who offered multiple opportunities for improving the management of CIs embracing a resilience-oriented research dimension. Several reviews are available in literature about resilience, but still restricted to specific CIs (e.g. energy systems (Gasser et al. 2019), cyber CIs (Mohebbi et al. 2020), remote sensing (Veettil et al. 2020), supply chain (Golan et al. 2020)), or with an explicit focus on quantification (see e.g. Hosseini et al. 2016; Ingrisch and Bahn 2018; Shuang et al. 2019; Rehana et al. 2020)). However, to the best of our knowledge, there is no explicit up-to-date literature review related to qualitative (or semi-quantitative) methodologies as developed for managing resilience of CIs.

The purpose of this study is to complement available literature in the context of CI resilience, through a systematic review on qualitative aspects, i.e. reviewing approaches explicitly focused on Tier I methods, or used as entry points for Tier II approaches (Linkov et al. 2018).

This paper reviews current literature on qualitative approaches used to manage and improve resilience of CIs, through firstly bibliometric findings and then via an in-depth descriptive content analysis. Our primary aim leads to explore and in-depth understand the diverse approaches available to support decision-making for CIs, and draw common concepts that emerge in case of the application of qualitative approaches. The research describes the hypotheses, methodologies, and results of relevant papers, extracting relevant knowledge from thematic full-text analyses which is intended to support scholars dealing with CI’s resilience mainly from a Tier I perspective (Linkov et al. 2018). While the research inherently focuses on Tier I approaches, it also partly deals with Tier II approaches, at least in those cases where the semi-quantitative dimension of analysis is explicitly combined with qualitative data sources.

This research has been conducted taking Scopus as a reference database, and then following a systematic review process based on the well-established PRISMA (Preferred Reporting Items for Systematic Reviews and Meta-Analyses) framework (Moher et al. 2009b).

The remainder of the paper is organised as follows. Section 2 details our research method; Sect. 3 illustrates main bibliometric findings; Sect. 4 discusses in detail each included paper; Sect. 5 offers points for discussion and insights from the literature review. Finally, conclusions on this work are summarised in Sect. 6.

## Methodology

**Fig. 1:**
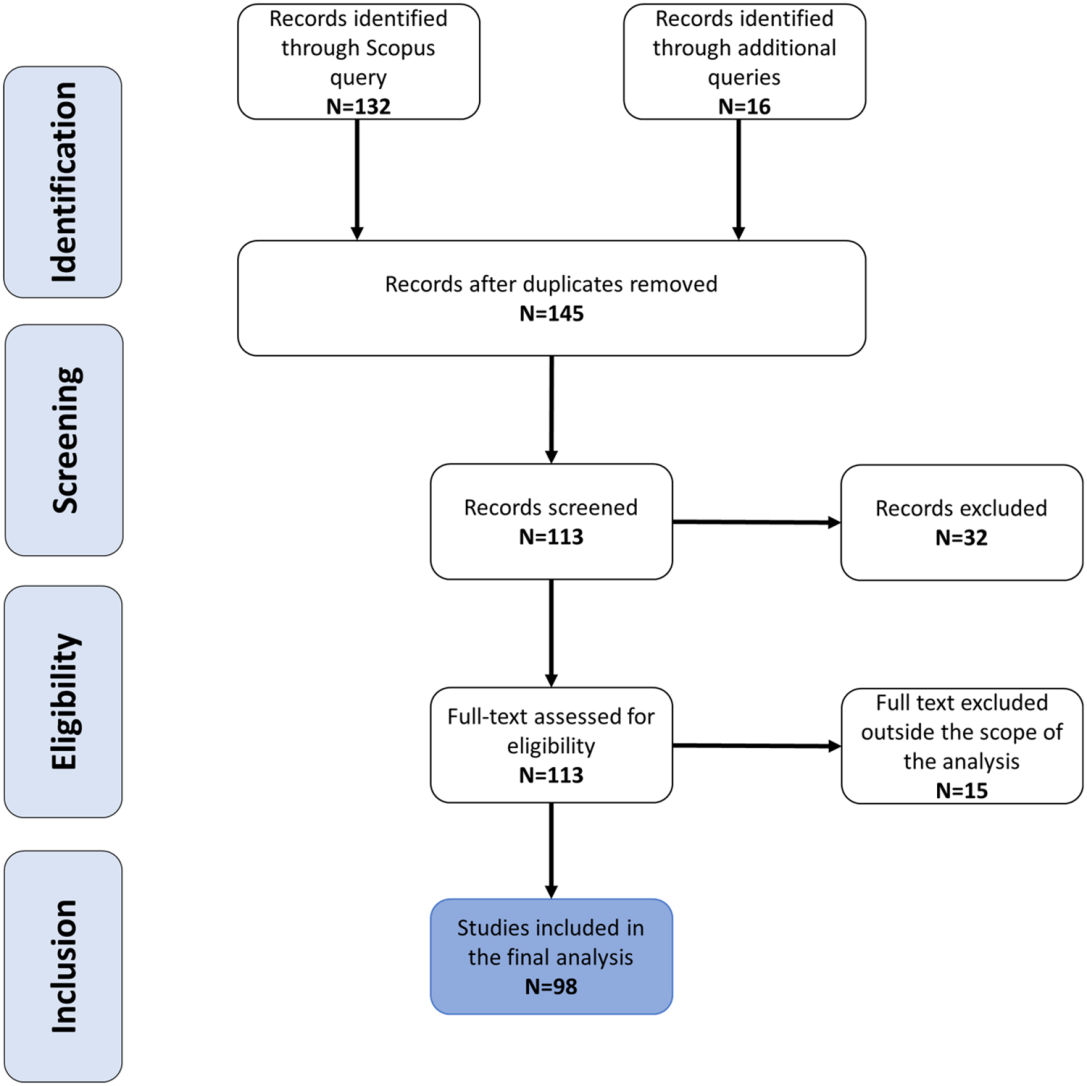
Literature search strategy

The systematic approach followed in this research relies on PRISMA (Moher et al. 2009a) and consists of 5 phases, as sketched in Fig. 1, conducted by authors of the article through the usage of MS Excel and Mendeley.

### STEP 1: setting the search query

The first step of the review defined the scope of the search query. A query has been progressively refined to include a large set of contributions potentially linked to qualitative approaches for CIs’ resilience. The final search query implemented in Scopus query system included every paper making use in title, abstract, or keywords of ‘resilience AND “critical infrastructure”’, as well as any of the following lemmas: interview, focus group, workshop, questionnaire, surveys, or the term qualitative. The selection of terms was inspired by (Patton 2002).

A specific inclusion criterion refers to papers indexed up to March 2020. The query has been limited to the Scopus database, since it is recognised as the biggest repository of peer-reviewed literature with over 5000 publishers and over 71 million records fairly balanced among technical and social aspects of science (Elsevier 2018).

Following an inductive process on abstract reading, the dataset has been enlarged to include other documents as referred to EU/US-funded projects mentioned in the full text of papers as assessed for eligibility, running an additional query with for title, abstract, or keywords including ‘resilience AND “critical infrastructure”’ AND the name of the project (e.g. CIRMin, DARWIN, IMPROVER, NEXUS, Resilience Shift, SMART Measure Resilience) (Adini et al. 2017). The two queries return, respectively, 132 and 16 documents.

### STEP 2: refinement of dataset

Overall, the queries returned 148 items matching the search criteria. As a first step, a preliminary data refinement on the analysis of titles has been conducted to eliminate duplicates: 3 duplicates have been identified and deleted, meaning that 145 documents progressed to the next phases.

### STEP 3: screening

The output of this step consisted of selecting documents relevant for the scope of the work. Each abstract has been screened and several documents were excluded because they were just mentioning the word “survey” as a synonymous for “review” or “study”, not implying the actual usage of any qualitative research method for managing resilience of CIs. After this screening phase, 32 papers have been excluded, meaning that 113 documents require full-text assessment for inclusions. The analysis has been conducted by two researchers independently (RC, RP), leading to an agreement ratio of over 95%. Ambiguous situations have been solved conservatively, keeping those papers for full-text reading.

### STEP 4: eligibility assessment

This step represented the analysis of documents following full-text reading. Through this step, 15 documents have been excluded, being considered outside the scope of the analysis, and showed a set of 98 documents to represent the final dataset. The analysis has been conducted by two researchers independently (RC, RP, over 95% agreement). The few incoherencies have been solved via a group discussion involving also the third researcher (GDG). While reviewing the full text, additional papers have been added to the dataset as explained in Step 1 (as included in the 113 documents).

### STEP 5: analysis of papers included in the final dataset

The last step consisted of analysing the full text of the 98 papers. This analysis also aimed to refine the meta-data on the paper to ensure meaningful bibliometric analyses, and to follow an ad hoc protocol for systematic knowledge elicitation. The protocol included aim of the paper; domain being investigated; type of qualitative approach; causes and threats; method or model used; metrics/indexes used or defined. The dataset has been split between the researchers and cross-checks have been defined to ensure higher consistency. The analysis followed a deductive perspective with an unconstrained categorisation: iteratively adding different categories was considered possible within the bounds of the protocols, in line with inductive content analysis (Elo and Kyngäs 2008).

On the results of this categorisation, documents have been presented following different dimensions of resilience for CIs to facilitate the narrative dimension of the document (see Sect. 4). In practical terms, the adopted logic led to identify four resilience dimensions, namely “techno-centric”, “organisational”, “community”, and “urban” within which different qualitative approaches were utilised, several threats or hazards were identified, and many issues were discussed in order to improve resilience.

## Bibliometric findings

**Fig. 2:**
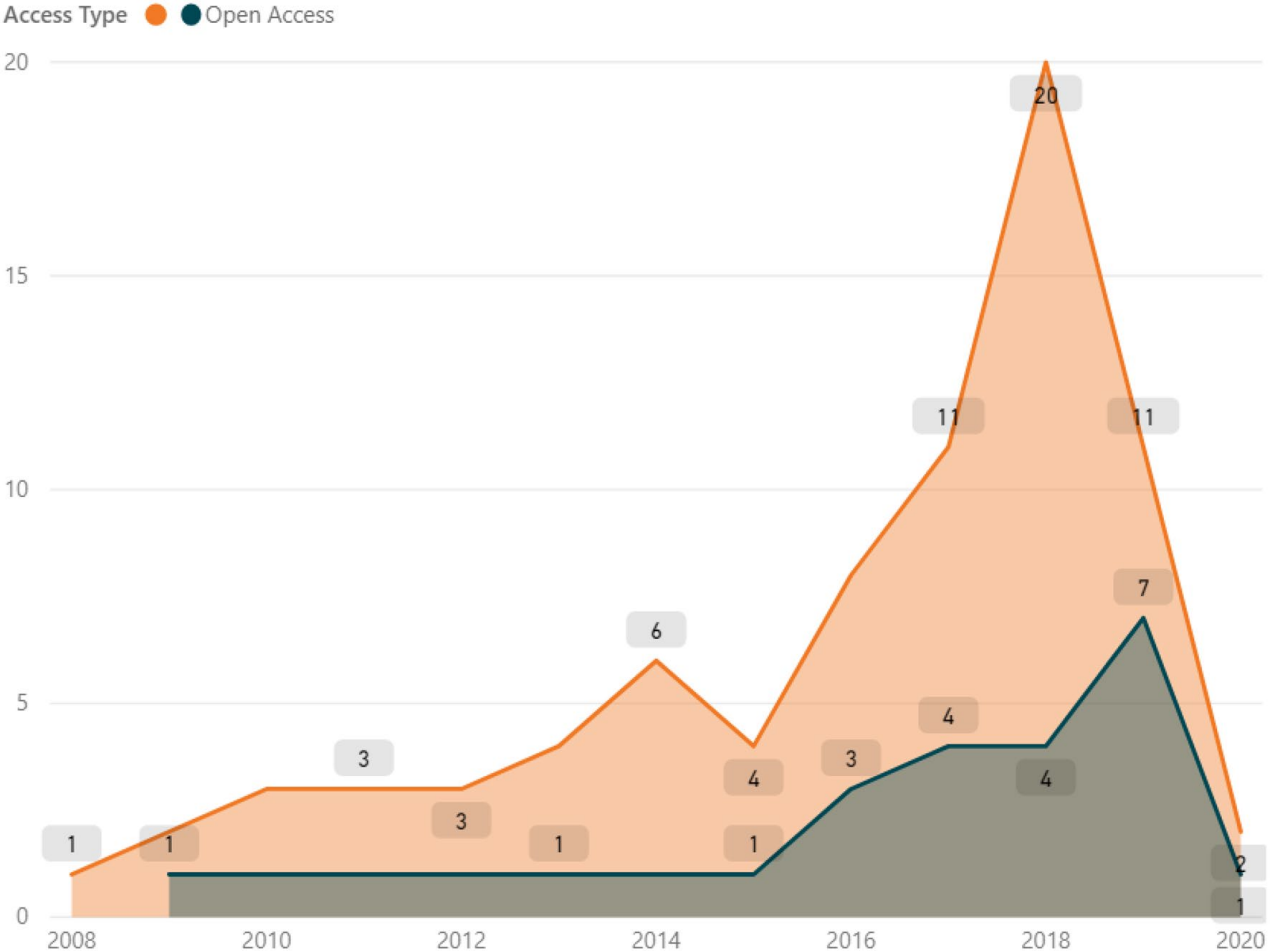
Evolution and access type of documents over years

In this section, some bibliometric findings are reported. Figure 2 shows the evolution of the examined papers over years, comparing as well open-access vs subscription-access papers. In this context, the increase of open-access documents in recent years is noteworthy, even though still below 40%.

**Fig. 3:**
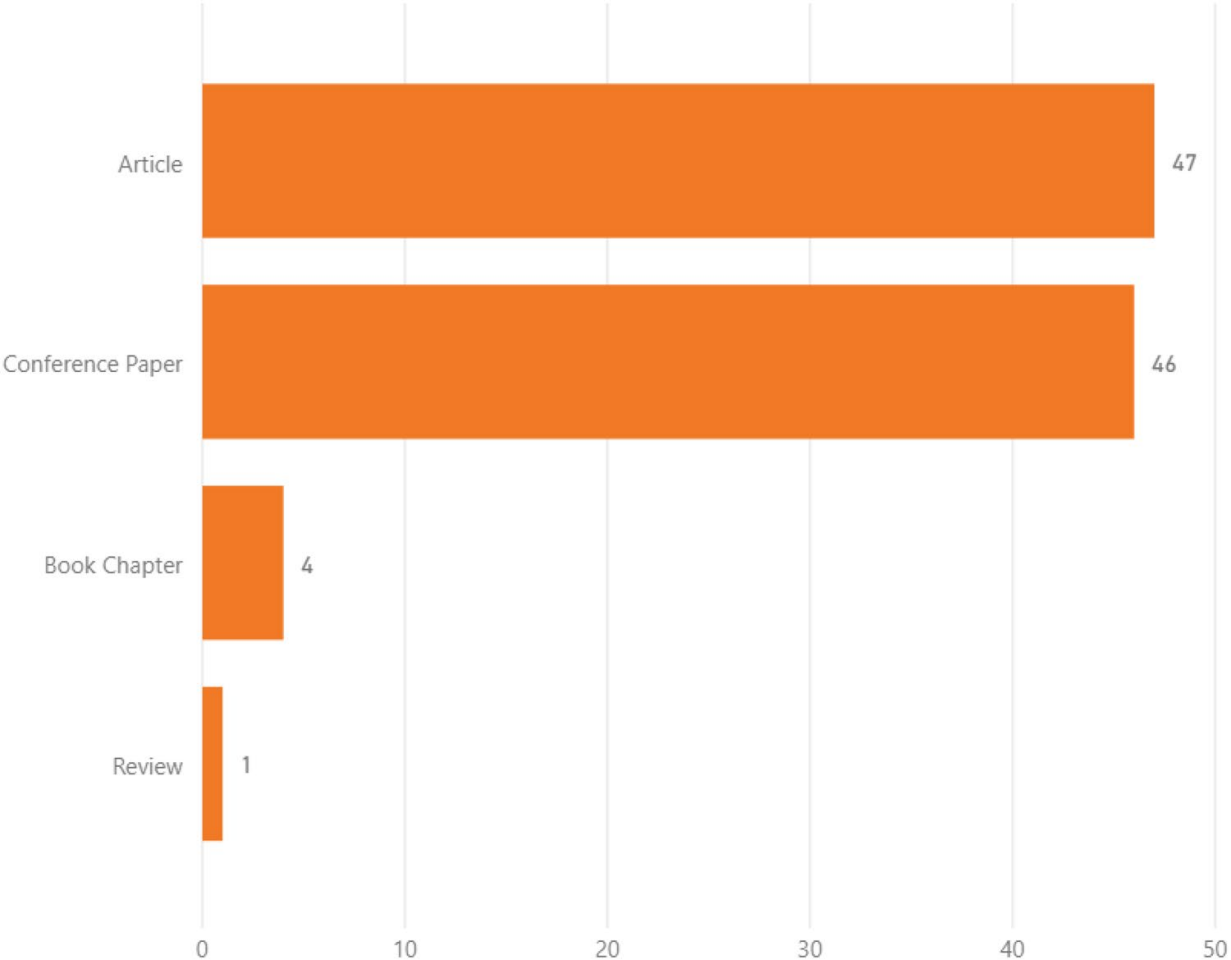
Document types in the dataset

Figure 3 considers the document types of the dataset and shows a balanced proportion between conference proceedings and journal articles. This means that when looking at CIs’ resilience from a qualitative perspective, both journals and conferences seem to be relevant publication fora. It is also worth mentioning that many of the proceedings are related to research conducted under various stages of funded projects, proving that conferences represent a preferable way for disseminating intermediate results.

More specifically, the publications are spread across different journal and conferences with a few sources including more than three papers, i.e. International Journal of Critical Infrastructure Protection (4 documents), International Journal of Disaster Risk Reduction (4 documents), International Journal of Critical Infrastructures (3 documents), Reliability Engineering and System Safety (3 documents), Sustainability (3 documents). On the other hand, the most recurrent conferences are European Safety and Reliability—ESREL Conferences (8 documents), Information Systems for Crisis Response and Management—ISCRAM Conferences (7 documents), and International Development Research Centre—IDRC Conferences (3 documents).

**Fig. 4:**
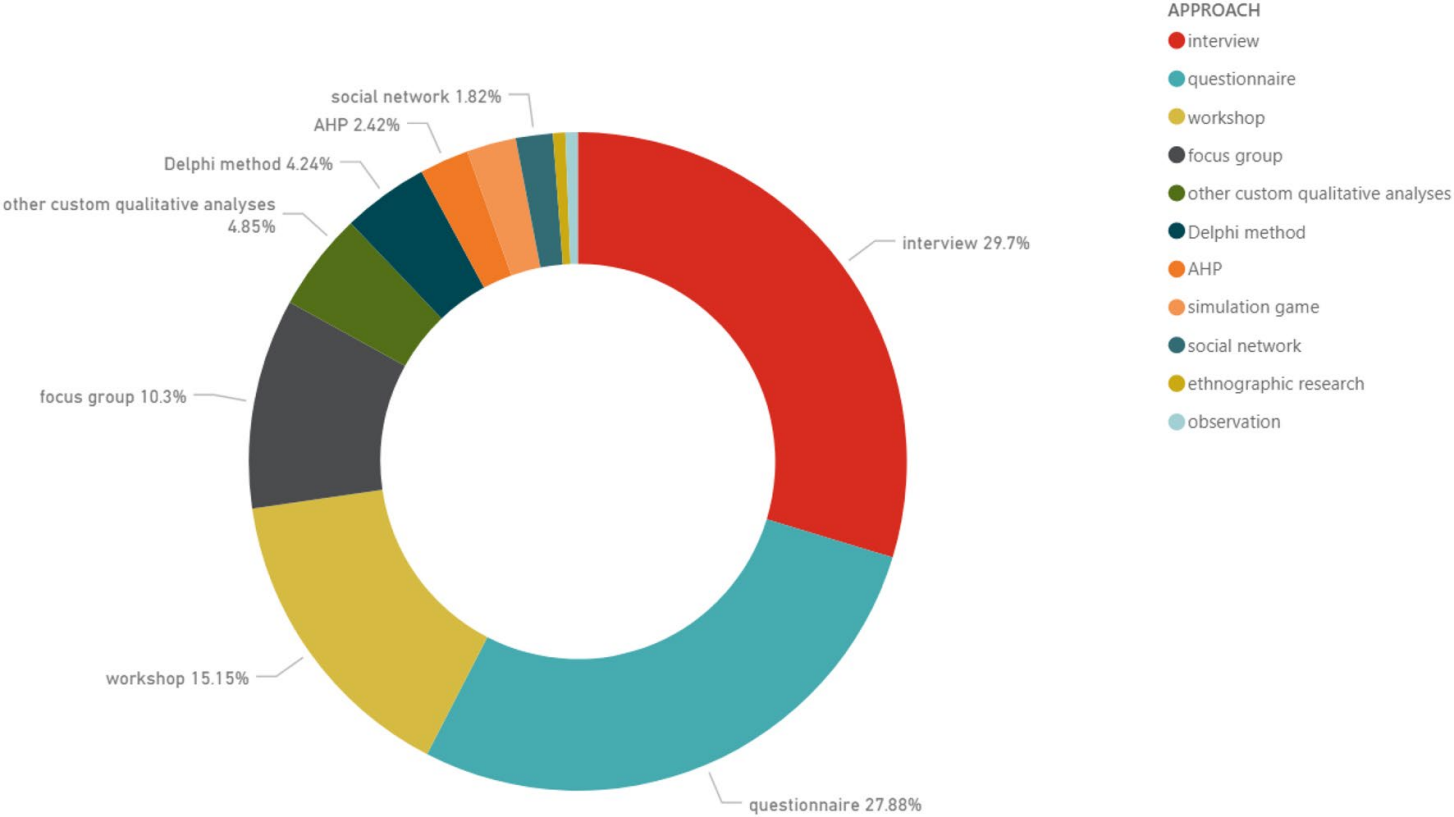
Percentage of used qualitative approach

More in detail, additional reflections can be proposed about the content of the papers in the dataset. Figure 4 reveals that more than half of them consist of interviews (about 30%) and questionnaires (about 28%). It is also relevant the percentage of approaches consisting of workshops (15%) or focus groups (10%), methods where experts or stakeholders can share their visions, opinions, ideas (focus group) or can find solutions and reach conclusions (workshops). Moreover, specific knowledge elicitation methods such as the Analytic Hierarchy Process (AHP) or the Delphi method have been utilised for prioritising concepts, ideas, attributes, guidelines, etc. These approaches remain of interest for qualitative research because of their knowledge elicitation process, which is at Tier I, qualitative by nature. Furthermore, simulation games have been discussed about their potential in engaging people and understanding human behaviours. Other sources refer to social networking, or some type of observation (naturalistic), or ethnographic research.

**Fig. 5:**
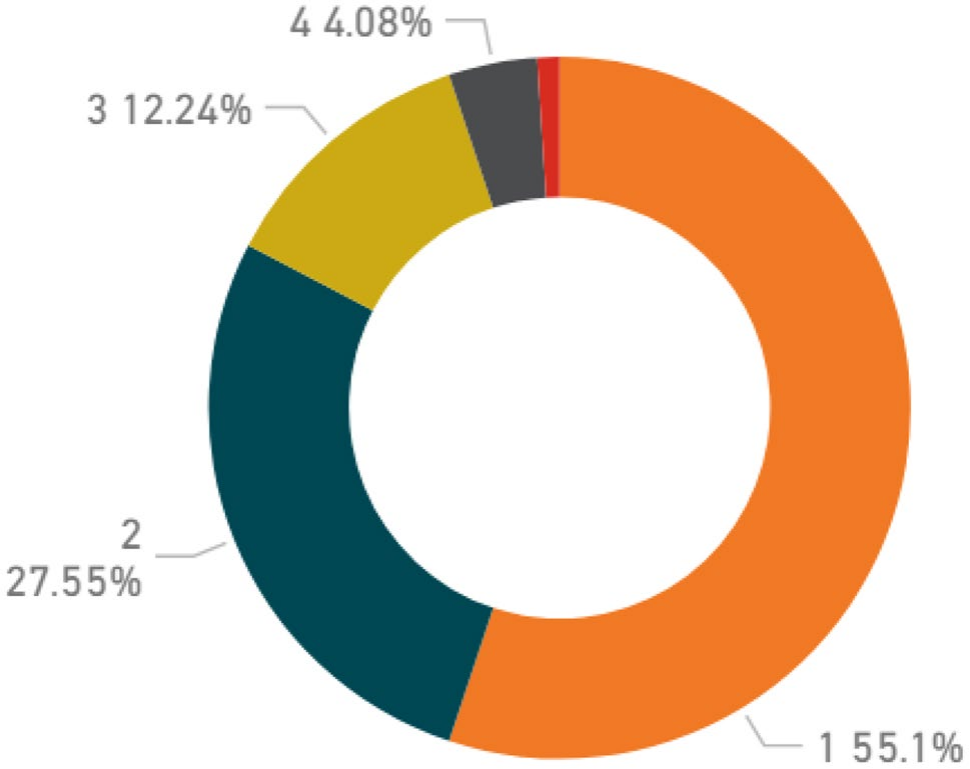
Number of different qualitative approaches utilised by each paper

Besides the overall percentages shown in Fig. 4, Fig. 5 shows the number of different qualitative approaches utilised among each paper. Although most papers (55%) apply a single approach, it is worth mentioning that the other half of papers utilises more than one approach, with about 20% of the documents using 3, 4, or event 5 techniques jointly. This observation might indicate the benefit of mixed-method research, and for the emerging need of knowledge triangulation.

**Fig. 6:**
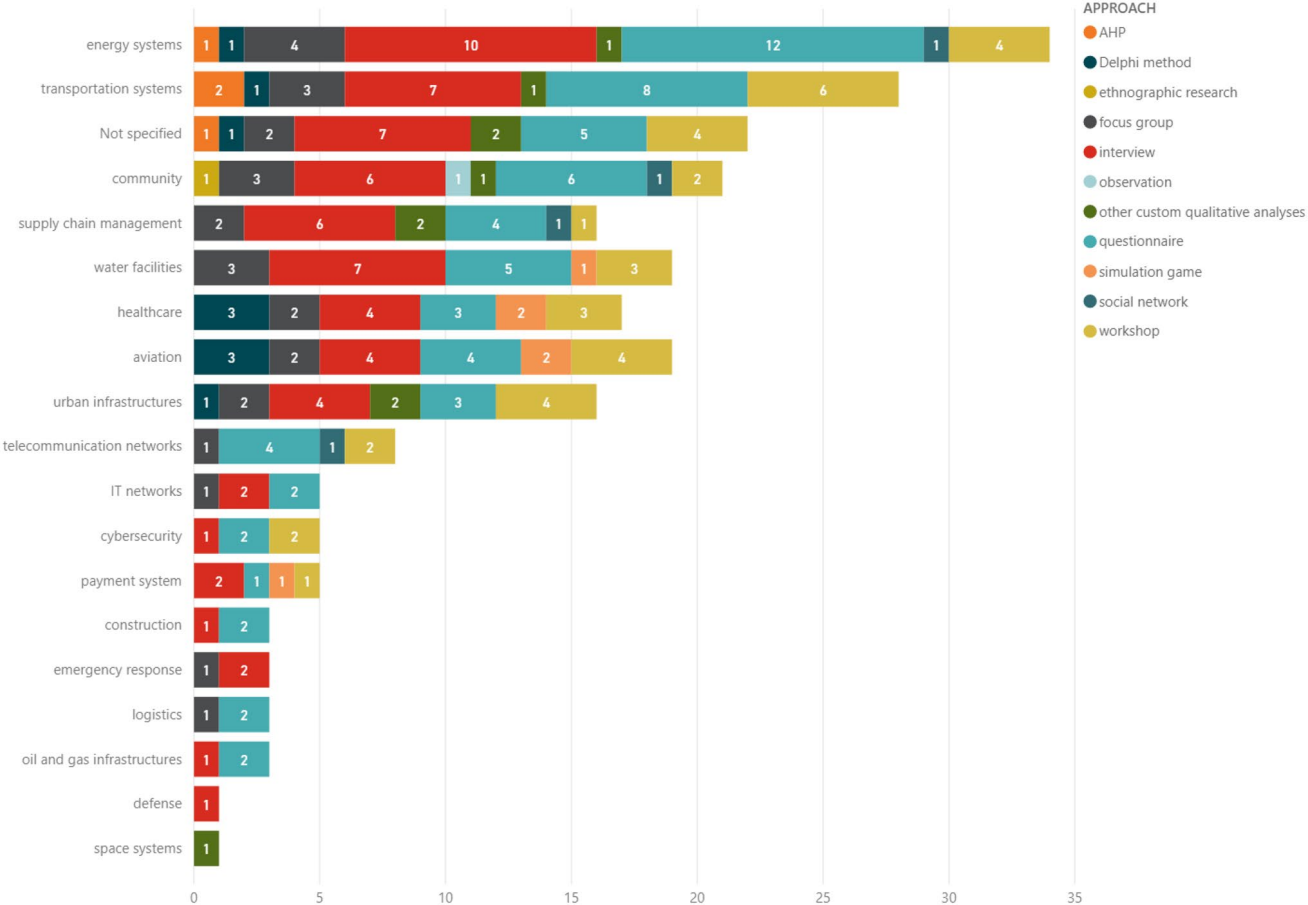
Different domains combined with different qualitative approaches utilised

Furthermore, the histograms in Fig. 6 show the different domains covered in the papers, combined with the typology of qualitative approaches utilised. The most frequent domain is related to energy systems (frequently ascribed to power grids), followed by transportation systems (e.g. roads, motorways, railroads, or ports). It can be also highlighted a non-negligible number of “not specified” documents because many papers do not refer to a domain or CI and do not contextualise their work, which therefore remains applicable to multiple domains. Other relevant domains are linked to community and urban infrastructures. Other CIs appearing in the dataset are supply chain management and water facilities.

Lastly, a combined view on the results is proposed in Table 1, which summarises the previous results: by rows, the figure offers the total of papers per domain, whereas, by columns, the total of papers per approach.

**Table 1:**
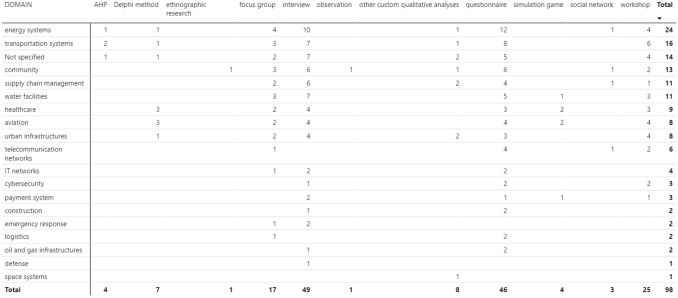
Overview of results

## Descriptive findings

This section proposes four different dimensions relevant for qualitative research applied to CIs, in line with recent research (Labaka et al. 2014). These dimensions encompass diverse, sometimes complementary aspects of resilience, i.e. techno-centric, organisational, community, and urban. The following sections are intended to provide a synthesis of reviewed studies, highlighting methodological steps, and obtained results. While these dimensions can be sometime overlapping, they are considered helpful to support the narrative understanding of the reviewed approaches, as further detailed in the Appendix.

### Techno-centric resilience

This paragraph deals with papers encompassing a research dimension that follows primarily the technical aspects of resilience with respect to several complex systems from civil infrastructures towards supply chains.

Among the wide range of CIs, e.g. water supply systems, sewerage, transportation infrastructure, power grids, and telecommunications networks, Sircar et al. (2013) focus on UK energy and transport infrastructures, via a set of scenario-episodes to support key stakeholders in the examination of their resilience. After an analysis conducted through the Political, Economic, Social, Technological, Environmental, Legal (PESTEL) lenses, tailored crisis episodes were added to stress test the scenarios and to operate in a not “business as usual” situation and a first set of key stakeholders from local resilience forums, engineering firms, UK Government departments, academia was interviewed to elaborate two tailored episodes related to flooding and terrorist attack, and other stakeholders were then invited to participate in focus groups, in order to consider strengths and weaknesses of the “2050 world”, guided by the three generations of resilience (cf. introduction).

When considering strengths and weaknesses of a region, it becomes crucial to focus on CIs’ spatial planning at different administrative levels as proved by a research conducted in Germany (Riegel 2014). Spatial planning considers and integrates the needs of multiple stakeholders, through the “mutual feedback” principle, which is an iterative top-down/bottom-up process to develop visions and principles of spatial development, to examine the compatibility of proposal for plans and projects, and to monitor impacts of realised developments on natural assets. A survey among all 111 German regional planning authorities examined the current perception of CI protection to increase their resilience. Even though the topic was generally known among the respondents, the survey showed how regions were not prepared to apply a systematic CI protection strategy as publicised by national security authorities or by European institutions.

In Canada, Valiquette L’Heureux and Therrien (2013) aim at assessing the main tendencies of the different dimensions of resilience within three major CI networks, i.e. transportation, energy, and telecommunication through a survey among hundreds of CI managers from governmental, community, and private-sector organisations who were identified screening official websites, annual reports, and official government information. The survey examined various aspects of internal and external resilience adaptative management, emergency preparedness, and post-crisis organisational learning. Resilience was assessed through a wide range of indicators using a Likert scale^1^ and the general attributes were analysed to seek statistically significant differences between groups of organisations by sector, type, and size (using Chi-square and Kruskal–Wallis). The empirical study provides insights into the main challenges and barriers to resilience: projective intelligence, decision-making abilities, strategic positioning, monitoring of risks, crisis management informational designs.

These challenges spans over technology-based, human resource-based, procedure-based strategies to be implemented for infrastructure resilience. The selection of the most adequate strategy becomes a multi-dimensional problem, as investigated through multicriteria decision-making techniques such as the AHP. At a higher abstraction level, the results obtained through a pool of experts from the Romanian Land Forces Academy suggested that the best strategy to increase the resilience of a CI refers to human resources, relying on an intensive training system of employees, in order to empower them within the system (Badea et al. 2017). Similarly, another research focused on the analytic network process (ANP), a variant of AHP, to prioritise factors affecting port resilience involving 11 experts including government officials, planners, and scholars. The consistency of ANP answers was ensured using the Delphi method leading to three major factors, i.e. ground access system, travel time, shipping route density (Hsieh et al. 2014).

Münzberg et al. (2017) aim at supporting building community resilience by introducing a spatial–temporal vulnerability assessment, based on various indicators, which enables decision makers (i.e. crisis management groups, management authorities, CI providers) to enhance their initial understanding of the impacts of a power outage. In this paper, the Delphi has been also adopted to ensure a consistent definition of an indicator for power outages, namely the Relevance Criticality Weight, as assessed by decision makers coming from different cities and disaster management authorities. This indicator has been combined with the Coping Capacity Resource (CCR) estimating the capacities of a CI to continue the activities during a power outage for a short time and finally integrated into a vulnerability model based on Monte Carlo simulation.

Other papers usually refer to Critical Infrastructures Key Resources (CIKRs) to describe infrastructure systems. In Vugrin et al. (2010b), the U.S. Sandia National Laboratories formulates a unifying framework which can be applied to all the CIKR systems identified by the U.S. Department of Homeland Security to study their resilience, explicitly considering the cost of recovery efforts. In particular, the discussed framework consists of an approach for quantitative measurement of resilience costs, and a qualitative method to evaluate features that determine systems’ resilience. The quantitative measurement involves two components: systemic impact (SI) which is defined as the difference between a targeted system performance level and an actual system performance after the disrupting event, and total recovery effect (TRE) which is the amount of the resources expended during recovery processes following the disruption. The framework also introduces a qualitative analysis, which can be performed to explain the quantitative measurement or can take the place of quantitative results when data are not available. This analysis utilises three system capacities to explain how inherent properties of a system can determine system resilience, by reducing SI and TRE. These capacities are absorptive, adaptive, and restorative. Better resilience systems can then be designed by developing resilience enhancement features that improve one or more of these capacities: for example, storage is an enhancement feature for absorptive capacity; emergency generators enhance the adaptive capacity; monitoring systems for break detection in power grids increase restorative capacity.

In Vugrin et al. (2010a), the authors utilise a previous framework elaborated in Vugrin et al. (2010b), to analyse the resilience of a particular CIKR like the U.S. petrochemical supply chain during hurricane disruptions. In particular, the Sandia centre performed a comparative analysis by simulating disruptions with the National Infrastructure and Simulation Analysis Center (NISAC) Petrochemical Supply Chain Model. This latter consists of two primary components: the chemical data model (CDM), a database of domestic and foreign chemical plants, chemical productions, commodity flows, and chemical infrastructures, and the NISAC Agent-Based Laboratory for Economics (N-ABLE) microeconomics simulation tool that performs supply chain analysis. Within the N-ABLE, each agent-based enterprise firm is composed by supervisors, production workers, sellers, buyers, and strategic planners. When applied to CDM, the N-ABLE can predict economic impacts and loss estimates to be used for measuring the systemic impact of a petrochemical supply chain affected by a hurricane. In addition, N-ABLE can help to estimate the costs associated with the recovery and adaptation processes, which are crucial for calculating the TRE in a resilience analysis. The framework allows to determine resilient costs starting from the calculated values of SI and TRE, but it also allows qualitative assessments of attributes that enhance the supply chain’s absorptive, adaptive, and restorative capacities.

In Petit et al. (2012), a Resilience Index (RI), useful for assessing CIKR facilities, has been developed. The RI can assist owners/operators to compare their facilities to similar sectors sites and can help them to make better risk-based decisions. The data were collected through a questionnaire of more than 1.500 data points per facility (i.e. commercial buildings, electrical substations, transportation assets, and dams). After a Quality Assurance review process, the RI was developed through an aggregation of data collected into four levels of information, by using multi-attribute theory, an approach that helped to decompose resilience into its individual attributes and then organised them into an organisational tree.

Trucco and Ward (2011) study the propagation mechanism and consequent dependencies of another CIKR, the Fast-Moving Consumer Goods supply chain of perishable goods in Italy. Fuzzy Cognitive Maps (FCMs) were utilised defining 13 concepts that reflected attribute, characteristics, qualities and paths of the modelled system, and tracing edges that represented the interconnections between concepts. The three independent FCMs for different temporal frames (i.e. after one day, four days, and three weeks) were implemented recurring to expert elicitation. Therefore, a specific questionnaire was distributed at least one week before, conducting a single face-to-face interview to define concepts and to weight the relationships in the adjacency matrices of the FCM approach. Regarding the case study, the results show that the most crucial concepts are the staff availability, the data network, and the electricity supply.

Also Seppänen et al. (2018) focus on the Infrastructure Failure Interdependencies (IFIs) developing a qualitative method, based on experts’ knowledge, for identifying and describing the potential sector-specific and cross-sector IFIs of CIs. The method, consisting of four phases (i.e. preparation, material collection, analysis, results), was developed and applied with the Finnish regional preparedness committees. In the first phase, the planning group defined the threat scenario. Then, company representatives from the studied CIs (i.e. electricity distribution, telecommunications, and IT infrastructures) were invited, together with representatives from other CI fields (healthcare and government agencies) in order to identify the broader interdependencies and to elaborate a scenario with a severe storm and a pandemic influenza. The second and third phase, respectively, material collection, and analysis, were conducted in iterative manner, performing a cycle which has been continued through several workshops with up to fifty experts, until the collected and structured material was satisfactory. In this case, the difference between the discussed method and the Delphi method is related to the anonymity of the experts: in the Delphi method it guarantees freedom of opinions and minimises the effect of dominant characters, whereas the facilitated face-to-face approach of the discussed method promotes the cooperation among the actors who would cooperate in the crisis situation. The discussions were recorded, and the collected information was first structured in mind maps and then the resulting documentation was used in the creation of an ISFI matrix that describes the causal intra-sector and inter-sector interconnections. Finally, the results were presented in system diagrams, a form that makes it easier to understand the failure interfaces between the CIs and the chains of dependencies.

As regards IFIs, Bloomfield et al. (2017) present a methodology called Preliminary Interdependence Analysis (PIA), useful for building, refining, and analysing models of interdependent complex CIs. This method starts with a qualitative phase during which the scenario must be accurately defined, and subject matter experts are asked to provide data and information which will be utilised to parametrise the selected model. Then, via a set of focused refinements, PIA may evolve into a quantitative method for assessing the risk due to interdependencies between CIs.

In order to analyse IFIs of specific supply chains and demand nodes, Pfeiffer et al. (2017) present a Grassroots Infrastructure Dependency Model (GRID-M). This model helps public safety officials to make better approximations about disruptions by using pre-incident survey data. GRID-M can also help the officials to gain near-real-time situational awareness on the physical state of a node by using the damage assessment application. GRID-M displays all outputs within a Geographic Information Systems environment with additional prepopulated layers such as real-time traffic and demographic information of the affected communities. This information can support the prioritisation of infrastructures in planning, exercise simulations, real-word situations, and restoration activities.

Beheshtian et al. (2017) use information extracted from geodatabase, applying the concept of network resilience to the motor-fuel supply chain (FSC) management in New York City, affected by hurricanes. The authors use a stochastic bi-stage optimisation model to analyse practical strategies for the allocation of resources, called Resilience-enhancing strategies (RES). For modelling the transportation network, an application program interface (API) implemented in Visual Basic was used to extract data from the ESRI geodatabase and translate them to a graph network consisting of nodes and arcs; then the impact of hurricanes were modelled by considering three characteristics: type and frequency; vulnerable locations; expected flooding intensity. The authors defined a context-specific resilience index referred to the average demand met across the gas stations, along with a variable representing the unmet demand rate (UDR), which allowed the authors to solve the model for the minimum expected value of the UDR, minimising the FSC’s overall inoperability and fuel distribution costs. Several experiments were conducted, varying policy scenarios, physical improvements, and budget limitations. The results showed how the three pillars (absorption, adaptation, and restoration) of the mentioned RES are interlinked and how different combinations of investment scenarios may provide different levels of resilience.

As discussed earlier, several threats can undermine systems included in the techno-centric dimension of resilience. Cutts et al. (2017), for example, deal with seismic risk. During the 2014 Cascadia Earthquake Readiness Workshop in Washington, the attendees were coordinated by facilitators in order to identify the most important infrastructure, related to three thematic areas (ports and waterways, critical energy infrastructures, and emergency management) exposed to the risk of an earthquake or tsunami and to brainstorm potential solutions. The participants produced a list of takeaways, observations, recommendations, and highest priority infrastructure improvements, to cope with possible earthquakes or tsunami and to increase infrastructure resilience.

Feldpausch-Parker et al. (2018) present a case study involving three different States (New York, Massachusetts, and Vermont) of the U.S. subject to the Sandy storm of 2012. The aim of this study is to compare how climate and energy are being linked in smart grid planning and development. Discussions from 22 focus groups included 3–8 representatives of a single organisation were recorded, professionally transcribed, and coded with NVivo 10.0 software to characterise discursive patterns and linkages between climate change and energy by analysing text focused on both climate mitigation and climate adaptation. Data analysis was based on the principles of grounded theory, which offers an internally logical set of techniques for collecting and analysing qualitative data. The triangulation of knowledge, via clarification questions and informant validations, supported a high level of accuracy, through a continual movement between data collection and analysis. Comparatively, the study found that the balance between the conversations about adaptation versus mitigation was associated with the severity of the storm’s impact in each of the three states. Differences between stakeholders were also examined, revealing that energy system operation experts (i.e. utilities) preferred adaptation measures to mitigation ones, while the opposite was for other energy system actors (i.e. authorities, researchers).

Matsika et al. (2016) introduce terrorism, as a man-made threat, requiring the development of risk assessment specifications for the RAMPART (Risk AssessMent toolbox for the Prevention and reduction of terrorist Attacks on metRo and light-rail criTical infrastructures) project. The paper focuses on the incorporating resilience in the risk assessment techniques. During a workshop with experts, six risk/resilience assessment methodologies used in Canada, EU, and USA were selected for detailed analysis and six key factors that constitute a robust risk assessment methodology (RAM) were identified. About one of these factors, “qualitative vs. quantitative approach”, the paper argues that a risk assessment in the public transport security should be done firstly in a qualitative way because of the lack of statistics (especially concerning terrorist incidents), necessary to perform a quantitative assessment based on mathematical formulas and calculations.

McBurnett et al. (2018) aim at demonstrating the effectiveness of simulation gaming for developing systems-thinking skills which are critical to understand the complex nature of infrastructure management. Using Vensim software, the authors implemented a system dynamic model to represent, through a simulation game called LA Water Game, the problem of maintaining the quality of Los Angeles water distribution infrastructure over a 75-year period. The game was performed in 16 workshops of over 200 participants consisting of undergraduate and graduate students, faculty, and active-duty military personnel. Data collection, through participant observations and debriefing interviews, showed the success of this particular teaching method: the players, with cognitive and effective engagement and intrinsic motivation, were able to identify the interdependencies between game variables, the non-linear nature of infrastructure deterioration, the stochastic emergency breaks, and the reinforcing loops within the game. Therefore, simulation gaming can be considered successful for training resilience skills.

Among techno-centric resilience, specific attention has been devoted to those systems with a large Information Technology dimension (Haass et al. 2015). For example, Johnsen et al. (2009) aim at assessing the safety, security, and resilience of Information and Communication Technology (ICT) and Supervisory Control And Data Acquisition (SCADA) systems used in the Norwegian oil and gas industry through surveying 46 Norwegian offshore oil and gas installations. In the survey, Yes/No answers were possible, but respondents could also provide free-form comments. The survey and the subsequent discussions provided some important key results: poor risk awareness, lack of consistent safety/security guidelines; absence of systematic knowledge sharing, poor scenario training, and emergency preparedness; lack of systems certification; lack of network barriers; inadequate deployment of patches.

In Landegren et al. (2018), a research is conducted to investigate the extent to which a simulation-based approach can be applied to large socio-technical IT networks. These networks (i.e. a municipal IT network and the SCADA system of a wastewater network) are modelled using Monte Carlo simulation to understand their recovery times. The utilised model is hybrid and considers the technical network, represented with graph theory, as well as the repair system, represented with a queuing model with four types of entities: jobs, queues, stock, and repairers. Data were collected through interviews with focus groups and through document analysis to gather information about fault modes, their relative probabilities, repair times, and resources needed for repair. The results were evaluated through interviews to check trustworthiness, usefulness, ability to increase system resilience, improvement, and generalisation possibilities. The authors conclude that this approach can be extended also including software and dependency failures to hardware and operator failures and considering investments, price of network, and repair resources as suggested by system experts’ feedbacks.

Dealing with Industrial Control Systems (ICS), Lykou et al. (2019) provide a review and analysis of available cybersecurity Self-Assessment tools, which can be utilised by ICT owners and CI operators. In order to identify weaknesses and cyber vulnerabilities and to establish targets for continuing improvement, these tools provide an assessment for many purposes: (1) they can be used by management teams to gain a general understanding of security assurance and make informed decisions; (2) they can be utilised as a guide to assess the status of security for a system; (3) they can enhance and support employees’ security awareness; (4) they may be used to fulfil reporting requirements, to prepare for audits or to identify resource needs.

Again on cyber vulnerabilities, Rajamaki et al. (2018) present the work in progress in developing cybersecurity and training in healthcare facilities. In particular, they focus on the Proactive Resilience Educational Framework (Prosilience EF), whose goal is to reduce cybersecurity vulnerabilities and exposure in hospitals and to make hospitals more resilient for cyber-attacks by identifying potential cybersecurity vulnerabilities and cyber threats; describing best practices and training for targeted and untargeted attacks; raising awareness of security and privacy of healthcare staff; developing training schemes on cybersecurity in hospitals for different categories of staff. The framework launches an iterative process of awareness and training development with relevant stakeholders (healthcare authorities, end users-hospitals, industry members, cybersecurity training providers), evaluating the framework via joint exercises and workshops.

Bernroider et al. (2016) aim at developing and testing a framework that holistically measures the quality of Information Security Management (ISM) in the context of cybersecurity and allows for comparative assessments of organisations in CI sectors, taking ideas from the Balanced Scorecards (BSC) measurement system. Following a design science approach, workshops, cyclic refinements of the instrument, pre-tests, and framework evaluation within 30 critical infrastructure organisations were conducted, involving subject experts and Chief Security Information Officers (CISOs) as the main stakeholders from the information security domain. The quantitative evaluation served to benchmark, but to complement this assessment by explaining and interpreting scores, open qualitative questions were necessary to capture the special context situations. The authors argue that the scorecards, used as quantitative estimators, alone do not portray the complete security status.

Student et al. (2018) present an indirect measurement method to improve preventative maintenance and increase resilience of CIs as communication networks or electrical transmission infrastructure. The authors start from considering that many instances of failed infrastructures are not immediately discovered by CI operators or owners, but rather, by the public who report the problems with relative and qualitative descriptions. The idea of the paper is to enhance these qualitative descriptions by using a human-in-the loop algorithm, derived from the concept of Agile IoT, providing quantitative measurements (through actionable intelligence from the general public who utilises own devices or by using already deployed sensors, like traffic cameras) which could help prioritising repairs to reduce the likelihood of failures and to better allocate time and crew resources.

In order to analyse resilience of a networked system that depends from ICT, like the Internet, Garcia et al. (2010) present a hardware-based emulation (emulation testbed). The authors study the fidelity of the emulation by comparing experimental results between two different emulation configurations against the reference real configuration. While confirming the efficiency and similitude of emulation testbed, the authors recommend that the interpretation of experimental results should not be based on absolute numbers, which are hardware dependent, but rather on system behaviour and trends. This means that emulations are representative of real systems from a qualitative point of view, in terms of emerging behaviour, rather than a quantitative perspective, in terms of absolute performance.

Reilly et al. (2018) explore how CI resilience can be enhanced through the information-sharing practices of its operators during each stage of an incident (mitigation, preparedness, response, recovery). Effective crisis communication should manage information through the collection and dissemination of crisis-related information, while also managing its meaning to persuade the public in the hope that they will plan for and respond appropriately to risks and threats. Interviews, focus groups, and consultations were conducted with 31 relevant stakeholders across Europe. CI and emergency management professionals were asked about current communication strategies, whether digital media had been incorporated, how traditional and digital media were used together, what audience they hope to reach using different platforms. Interviews with journalists focused on their experiences of social media in detecting and verifying incidents, and whether they addressed ethical and legal challenges, using social media in relation to emergencies. The public indeed expects CI operators to keep them informed about progresses on restoration of CI infrastructures and answer rapidly to queries, as proven by a large questionnaire-based study (N = 403) and several semi-structured interviews with multiple stakeholders (Petersen et al. 2020a).

Finally, Gheorghe et al. (2018) deal with the resilience of particular ICT systems which are subject and object of crisis and emergency situation management: Critical Space Infrastructure (CSI), like satellites orbiting the Earth, may compromise, with their failures, the capacity of competent actors to manage the crisis. As regards the space governance, the authors argue that space actors must agree on key resilience measures, implement them and enforce them unilaterally on third parties, such as corporations or other states, despite the lack of jurisdiction due to the international character of the space environment. Moreover, they foster the cooperation among space actors and the interoperability of systems, to ensure timely access to various resources or to substitute one space system for another in terms of short-term provisioning of critical space services.

### Organisational resilience

When dealing with CIs, there are usually non-negligible organisational components to be considered in the analysis, i.e. components which embrace a social dimensions complementary to the more technical aspects of a system. This section emphasises on the contributions which make an explicit effort to understand, model, and measure the organisational resilience for CIs (Pathirage 2010).

Referring to construction organisations, Sapeciay et al. (2019) identify strategic resilience indicators through a triangulation analysis of literature review, questionnaire survey, and in-depth interviews. Firstly, an extended literature review revealed 72 indicators related to organisational resilience, among which 27 indicators were selected according to their frequencies, by means of Nvivo software. Secondly, an electronic questionnaire was undertaken to elicit the views of construction practitioners and resilience experts in New Zealand. The questionnaire was composed by closed-ended and open-ended questions and aimed at identifying the profile of respondents and organisations, at getting an overall view of their organisational resilience practice, and at finding out respondents’ opinions on key resilience indicators and ranking the latter ones. Thirdly, semi-structured interviews were conducted with 23 construction practitioners from construction client and contractor organisations (i.e. mainly project senior executives). The transcript interviews were qualitative analysed by using Nvivo software, searching for key themes. Triangulation of data improved reliability, by reducing judgmental bias, and supporting validation of the most significant indicators through interviews. Most experts agreed that the top five indicators for assessing resilience of the construction industry are, in rank order: leadership, planning strategies, internal resources, decision-making, and staff engagement.

Again in New Zealand, Brown et al. (2017) present a method to benchmark the organisational resilience of CIs’ providers, i.e. the Benchmark Resilience Tool, based on 13 indicators of resilience. Respondents were asked to use a Likert scale to rate how much they agreed with each statement and had to answer other demographic and preparedness questions (gender, age, organisation use of emergency plan, etc.). The study assesses relative resilience strengths and weaknesses of CI organisations, finding the “effective partnership” as the strongest indicator whereas “breaking silos” (i.e. breaking barriers to the sharing of ideas and skills) and “stress testing plans” (i.e. the capacity to actively practice emergency, crises, or business continuity plans) as the weakest ones. Findings also show that senior managers have much more positive perceptions of the resilience of their organisations compared with other staff workers.

Conducted by a private company, the study described in (Mendonça and Wallace 2015) provides a critique of standardised factors for organisational resilience, analysing the restoration process of electric power in Manhattan after 11 September 2001. Qualitative and quantitative data were collected to support triangulation of observations. Internal reports from the company and articles in the popular press provided also contextual information on the company’s response, while data regarding the timing, cues, and key decisions were provided through the Critical Decision Method (CDM). Data on participants were collected by questionnaires and data on the performance of technological systems (i.e. timing and location of restoration activities) were collected through logs of the performed work. The Woods factors (i.e. buffering capacity, flexibility/stiffness, margin, tolerance, cross-scale interactions) were analysed and the study proposed another factor—boundary-spanning capability—which may help to understand how cross-organisational linkages can help to determine organisational resilience.

Adini et al. (2017) present a work which is part of the European research project DARWIN, whose aim is improving responses to crises affecting CIs or social structures by developing resilience management guidelines (Cedrini et al. 2018). The first phase of DARWIN Project was to identify concepts, practices, and approaches of resilience management through a Systematic Literature Review and interviews with relevant stakeholders involved in crisis management. A final list of 56 concepts, practices, and approaches was compiled, phrased in a uniform mode, and incorporated into a computerised survey tool, using Survey Monkey. A 2-cycle modified Delphi process was conducted to decide which items of the list should have been included in the resilience management guidelines. Reviewing the items that reached the highest scores reveals that they comprise at least one of the three common elements frequently found in definitions of resilience: the need of flexibility, adjustability, and adaptability; the need for sharing and understanding for the actors involved; the focusing on CIs, considered vital for organisations and communities.

Förster et al. (2019) introduce another ongoing work within the scope of the DARWIN project: the resilience management guidelines. These latter, applied to the Air Traffic Management (ATM) and the healthcare domain, are intended to support organisations in critical situations and evaluate their effectiveness by attribute- and performance-based approaches, in line with, respectively, engineering resilience and resilience engineering (i.e. resilience in technical systems and resilience in complex socio-technical systems). Simulation trials, performed by operational experts in the form of gaming sessions, assessed the effectiveness of the adopted operational procedures. Additional scenarios were developed to emphasise the organisational interdependencies between ATM and healthcare CIs. Finally, a debriefing was conducted to assess the performance of the system, indicating possible bottlenecks and identifying brittleness.

Field and Look (2018) aim at assessing organisations resilience performance, providing a framework based on a value model, against which each type of CI was comparatively assessed. A set of interviews was conducted to 50 industry experts from a variety of CIs’ sectors with direct responsibility for the assess risk and resilience. Each infrastructure type was reviewed in terms of value expectations from its various stakeholders’ groups (i.e. end user/customer to investors, suppliers, and constituent organisations). The evidence for these assessments was based on the results of the interviews in addition to performance metrics published by government authorities, regulators, consumer organisations. Key findings were the lack of incentives to work proactively with other providers; the focus on response and recovery instead of proactive mitigation measures; the non-measurement of the impacts of disruption on UK society.

Based on the INTACT project co-founded by the European Union, Räikkönen et al. (2017) focus on Risk Management Measures (RMM). In particular, the study contributes to the value creation of RMM, which is crucial for decision-making and for the development of strategies to prevent or reduce the impacts of extreme weather events. A case study, regarding the electricity distribution network in Finland, was presented to validate the proposed approach. The stakeholder value of RMM during the entire life cycle of CI was assessed, by applying AHP to the considerations and evaluations made by 18 experts from the DSO (Distribution System Operator), the regional rescue service, the city of Tampere, the Finnish Red Cross, and an ICT company. The AHP method ranked the following value criteria for comparing the RMMs: benefits of the RMMs in economic, environmental, and social terms; impact of the RMMs on reliability, availability, and maintainability of electricity distribution network; life-cycle cost (investment and operating costs) of the RMMs. Then, the involved stakeholders were divided into three groups and they identified alternative RMMs which were categorised according to the phases of the disaster management cycle (i.e. mitigation & prevention; preparedness; response; recovery). The findings of the assessment ranked the following RMMs in order of relevance: mutual planning and training, underground cabling, ICT systems, and forming and disseminating situational awareness.

Another paper deals with climate change (CC) and its related natural hazards for CIs. Airports are CIs particularly at risk from the potential consequences of CC with impacts like sea-level rise, increased temperature, changes in precipitations or in wind patterns. The work presented in Burbidge (2016) starts from the studies carried out by EUROCONTROL, the European Organisation for the Safety of Air Navigation, to demonstrate that, although awareness is growing, there are still significant barriers to take actions (e.g. lack of reliable information, missing guidance). Therefore, EUROCONTROL, together with Manchester Metropolitan University, organised a workshop, attended by 30 participants representing industry, regulators, and academia, which led to identify four key priorities to overcome these barriers: better understanding of the problem; assessing the problem; initiating actions to adapt both operations and infrastructure; collaboration in research and information sharing and communication of best practices both within Europe and globally.

Taking into account the international framework to promote disaster risk reduction throughout the education sector, Bandecchi et al. (2019) focus on assessing the school resilience, from an organisational point of view. A survey, made by 7 different types of questionnaires for different ages (from 3 to 19 years) and respondents (students, headmasters, professors, auxiliary personnel), was conducted in 27 schools in Tuscany (Italy), located in areas of high geo-hydrological and seismic hazards. The questions were inspired by the Naylor examples of assessment techniques and were structured in different ways to identify criticalities in the community management process: closed question, open-ended question, completing table, matching exercise, cartoon strip sequence, graphic organiser, sequencing, graphic open-ended question, and graphic closed question. Shared results have been obtained from a resilience analysis on US campus emergency management units, by means of a survey, and then investigated in statistical terms (Murphy et al. 2019).

Shifting towards civil response management, Große (2019) examines the Swedish Emergency Response planning process, trying to identify and characterise the sources of uncertainty with respect to power shortages. This planning is a multi-agency activity that requires decomposition and coordination of goals and means throughout a multi-level approach. It involves local actors as municipalities and power grid operators, regional actors like county administrations, and national actors as agencies. The author conducts a literature and document review analysing guidelines, national laws, and reports regarding the Swedish description of the planning approach. Thereby, sources of uncertainty, stemming from lack of knowledge, emerged and guided the meetings with planners at municipalities and County Administrative Boards (CABs). Semi-structured interviews were then conducted, registered, and transcribed: a questionnaire with open-ended questions, based on the sources of uncertainty, was utilised to guarantee a similar structure for the interviews and to allow participants to report on individual experiences and perceptions. Follow-up questions were asked for more richness of detail and clarity. This study revealed three sources of uncertainty: the planning reference process in general, the decision-making process, and the direction and guidance alongside these processes.

In the same national context, Olausson (2019) deals with the resilience in the case of power shortage, focusing on the Swedish STYREL policy. STYREL (a Swedish acronym for “Steering of electricity to prioritised users during short-term electricity shortages”) is a planning and prioritisation process, involving national authorities, CABs, and municipalities. The aim of this process is to identify and prioritise the vital societal functions that must be carried out during a situation of power shortage, to foster sustainability and to increase resilience for power supply. A survey including 21 coordinators at the regional level and semi-structures interviews at three different CABs were conducted to provide a broad picture on the importance of the process, its usefulness, and the trust within the networks. However, according to the frank discussion outlined by the author, there are no guarantees that STYREL process, such as it has been carried out, will make the electrical energy supply more resilient and sustainable; nor does it seem that STYREL has created any formal or established type of collaboration between private and public sectors actors in practice.

Also Hiete et al. (2011) analyse the possible impacts of a power outage, focusing on the German healthcare. As effects and measures are strongly determined by the duration of the power outage, three scenarios are developed reflecting three different outage durations: below 8 h, between 8 and 24 h, more than 24 h. Discussions on these scenarios were fostered among the participants and three sub-groups were formed for the impact analysis. In a second step, Preparation, Mitigation and Recovery (PMR) measures were collected and discussed, and results were sent to experts for commenting. Finally, semi-structured interviews with additional experts were conducted to have a more balanced view. The analysis of impacts represents an important starting point for the identification of crisis and continuity management measures referred to the three different phases of the crisis management process: prevention, crisis management, and recovery. Another important result of the workshop was that good cooperation between administrative authorities and healthcare providers as well as power suppliers may reduce negative impacts significantly. Cooperation includes sharing of information, for example on the expected duration of the outage, on the patients needing power-dependent medical devices, and on respective resources.

Line (2013) focuses on power industry and examines how distribution system operators (DSOs) align their policies to the principles of resilience within information security. The author performs a case study of incident management, conducting 19 semi-structured interviews to survey current practices and to identify the improvements needed to enhance smart grids. The ISO/IEC 27,035:2011 incident management process scheme, composed by five phases (plan and prepare; detection and reporting; assessment and decision; responses; lesson learnt), is used to investigate findings from the interviews to ICT and power automation systems managers of six large Norwegian DSOs. The investigation reveals that DSOs have quite some steps to go in the direction of being resilient organisations with respect to information security: plans for responding to information security incidents do not exist in all DSOs, training is an underprioritised activity, noticing and evaluating minor incidents are not common in practice.

The energy sector constitutes a societal economic driver which requires to study its vulnerabilities, barriers, and resilience (Pathirage and Al-Khaili 2016). Among the personnel of three different power plant facilities (Abu Dhabi, Dubai, Sharjah), 42 questionnaires containing close-ended questions, "yes/no" options, and few open-ended questions were answered by professionals aged between 30 and 40 and semi-structured face-to-face interviews were undertaken with the top management. Questionnaire results were shown to respondents, who were asked to comment their own perspective, and then interpreted via a resilience management lens. Data were elaborated through MS Excel and SPSS, generating descriptive statistics. Findings indicate terrorism, atmospheric, and tectonic hazards as the main risks of vulnerability, while the lack of or absence of national government legislation, and awareness and education were revealed as the main barriers.

Carpenter (2014) aims at creating a picture of the strengths and weaknesses of the state of Georgia and Savanah area, based on a review of existing resilience initiatives and interviews with CIs’ representatives. About 30 individuals among facility managers, safety specialists and other representatives of Georgia-based CIs’ sites (i.e. state, local, military, and private facilities) were interviewed. Questions included current practices that enhance and detract from resilience, barriers to resilience, development of contingency plans, kinds of exercises conducted, identifications of less or more prepared sectors, identification of interdependencies and cascading effects between sectors, command structures, leadership, public outreach, how to allocate resources to best improve resilience.

With a similar focus, the framework presented in Labaka et al. (2014) provides a set of policies to be implemented in order to increase CIs’ resilience level. These policies were obtained through several research methods: Group Model Building workshops; examination of multiple case studies of different past major industrial accidents; complemented by a Delphi method where 15 multidisciplinary experts from different sectors (academic, transport, energy, and first responders) took part answering two different questionnaires with different aims and contents. The policies were classified based on the four resilience dimensions (technical, organisational, economic, and social) and on the two resilience types identified (internal resilience referred to a specific CI and external resilience associated to involved external agents such as government, first responders, and society). Finally, 25 experts were asked to provide the temporal order in which the policies should have been implemented, to achieve a high efficiency and effectiveness in their implementation.

Some papers start from disruptions in the payment systems and investigate the organisational aspects to increase their resilience. Berggren et al. (2018) come back on gaming simulation and aim at creating a game for participants from food, fuel, and financial industry sectors, who could benefit from major training to understand the challenges posed by interdependencies like a major disruption in the payment systems. In particular, the authors focus on the design choices for developing a mixed-methods approach to assess teams’ resilient capability. The authors suggest that team resilience can only be captured by a holistic mixed-method that considers both “soft” aspects like team workload, collaboration, trust, shared awareness, collected through verbal questions or queries, as well as "hard" measures of team qualities and performances, considering some indicators like payment options, good flows, trust, and security.

Also Van Laere et al. (2017) identify and discuss challenges faced in case of disruptions in the payment system. The method is based on inductive qualitative research. Data sources included documents of previous incidents, interviews with key representatives of each relevant sector and two workshops with local and national actors. Results from document study and interviews were utilised for building two scenarios discussed during the following workshops, whose outputs were analysed to identify seven challenges for CIs’ resilience. The analysis proves resilience to be not only a matter of technical measures (i.e. alternative payment solutions, rationing fuel or food, and offering services to the vulnerable part of society), rather involving several communicative challenges (i.e. maintaining trust, preventing hoarding, avoiding panic).

Another paper deals explicitly with the organisational aspects of the payment system: Johansson et al. (2018) aim at understanding how local businesses in the sectors of banking, food, and fuel distribution are prepared to manage any disruptions. Six semi-structured interviews were recorded and transcribed, and a thematic analysis was then applied to the transcribed material. The results show that food, fuel, and bank sectors are not prepared for a long-period disruption in the payment system. The respondents trust others (mainly IT Support) to solve the problems and they assume that disruptions last few minutes or hours. There is no plan, so the respondents are likely to shut down their business if the payment system is not working for a longer period. These concerns may be also emphasised in the current pandemic era.

### Community resilience

Following an even broader perspective, this paragraph illustrates those papers that deal with CI resilience at a community level. Resilience is interpreted here as the ability of a community to handle surprises, avoid disasters/accidents, and to be able to recover to a satisfactory state, i.e. normal societal operations (Johnsen and Øren 2015), and it is now discussed in terms of its respective qualitative research methods.

When dealing with a community dimension of resilience, it is necessary to take into consideration the role of public perception and public tolerance levels for minimum level of service and rapidity of service restoration, as demonstrated widely by IMPROVER project (Lange and Honfi 2017; Rosenqvist et al. 2018; Storesund et al. 2018; Grosse 2019). This latter indeed, adopting a wide variety of qualitative research techniques (e.g. questionnaires, interviews and workshops), provided evidence of the crucial role played by public engagement in the management of CI resilience (Petersen et al. 2020b). Among the experiments, it appears relevant the case study on the transportation system related to the Oresund crossing between Denmark and Sweden (Petersen et al. 2018), or the Hungarian highway (Petersen et al. 2019b).

Banks et al. (2016) focus on the resilience of a rural community of central Appalachia, a U.S. remote mountainous region prone to flooding. An inter-professional team of nursing, architecture, and engineering students conducted a comprehensive assessment of health and environmental living conditions via an ethnographic data collection approach, using both quantitative and qualitative sources of data: interviews, surveying, open-ended questions, observations, measurement, and photography. Qualitative interview data were organised and securely managed using NVivo software, while quantitative data were organised into a database and descriptive statistical analysis using SPSS 22. A model of resilience for the rural Appalachian community was developed, depicting the cycle of facing hardships, rebounding, supporting one another, and building community.

Also Mavhura (2017) focuses on building community resilience in the Muzarabani district of Zimbabwe, often subjected to drought and flood-related disasters. The study applies systems-thinking approaches to examine how rural livelihoods address such living difficulties. 40 interviews were conducted among local authorities, traditional leaders, villagers, and health officials, focusing on the five livelihoods pillars: Natural, Economic, Human, Physical, Social. Then, three separate focus groups were held on the same capitals, comprising people who experienced the highest magnitude floods in 2008. Finally, a survey involving 700 households was conducted through questionnaires self-administered with the help of research assistants. The results were analysed by using thematic analysis and descriptive frequencies in Vensim software. In the study, resilience emerges as sharing resources among flood and draught victims and shows existing absorptive and adaptive capacities that smallholder farmers put in place to cope with natural hazards. Ethno-based flood and drought warnings, temporal migration to highest zones, particular social net (*Zunde raMamb*) and scheme (*nhimbe*) to share resources: these are the constituents of absorptive capacities. Adaptive capacities like practice of flood recession agriculture (*mudzedze*), dual cropping system, traditional flood proofing structures (*dura* and *dara*) suggest that the community has also the ability to store and recall flood experience, to learn and reorganise resources to address flood threats.

Simpson et al. (2010) focus on CIs’ measurements, using recovery curves, after the impact of the hurricane Katrina in 2005 over two coastal communities of the Mississippi region. Data were collected by interviewing key informants as public works administrators, government officials, and emergency personnel. Photographic evidence, Geographic Information Systems (GIS) data, and public documents were also used to integrate interviews. Press releases and media reports completed the missing data, although these releases and reports are, according to the authors, inherently sub-optimal sources: press releases are issued from corporate sources and tend to put organisations in good light by minimising failures, while media reports look for the emotional and interesting issues. Therefore, the research underlines the importance of data collection in emergency, establishing recovery curves and standardised measurement technologies for infrastructure, as helping tools to understand the effects and assist community to prepare for next events.

Still on hurricane Katrina, Glavovic (2008) presents the vicissitudes of the city of New Orleans. The author visits the region six times after the disaster and conducted naturalistic observations and interviews with planners, academics, and other people involved in the reconstruction efforts. The research, conducted with qualitative methods, allowed developing a conceptual framework to define principles for guiding actions related to building more sustainable and hazard-resilient communities. In this framework, the task of the governance institutions is to mediate human access to natural resources and prevent human beings impacts from exceeding certain thresholds. Then, the authors provide a list of principles aimed at involving local communities in the decision-making processes and on the prioritisation of ecological sustainability.

A similar focus on the reconstruction process has been documented in Ong et al. (2016), but in Tacloban city (Philippines), after the typhoon Haiyan in 2013. The authorities decided to implement a No-Dwelling Zone (NDZ) along the coastline. Housing reconstruction and people relocation programs were started to move households to other safer areas. Among these people, the authors conducted paper-based questionnaire surveys to investigate disaster experiences; assistance received; reconstruction or relocation experience; household and community decision-making; demographic profile. The responses were triangulated with focus groups with beneficiary households and key informant interviews were conducted with government and non-government organisations. Finally, the collected data were examined by using Wilcoxon–Mann–Whitney test for categorical variables, Spearman’s Rank Correlation for ordinal variables, and One-way Analysis of Variance for comparisons of groups. The questionnaire survey was conducted at three different sites that used three different approaches: owner-driven on-site reconstruction; community-driven off-site relocation; contractor-driven off-site relocation. Household respondents were asked to indicate their level of satisfaction regarding the programs, based on a five-point Likert scale. In terms of resilience management, results showed that on-site reconstruction was delayed to insufficient assistance schemes like materials and skill training, while off-site relocation was delayed by prolonged land acquisition and subcontracting issues. Satisfaction levels of respondents were affected by disruption of CIs, such as water and utility services, lack of livelihood opportunities as markets or business establishments, and proximity to learning facilities.

Herrmann Lunecke (2015) deal with the Chilean response after the severe tsunami of February 2010. After examination of the Chilean law framework for coastal planning, the study analysed tsunami mitigation measures and policies developed at local level in recent reconstruction plans. Then, a total of 50 semi-structured interviews and questionnaires with key local and regional actors of public and private sectors, as well as community leaders, were conducted to analyse tsunami impact mitigation measures. Processing and analysis of the collected data were realised through a simple content analysis and a frequencies analysis of each response category. This study found that mitigation measures in Chilean coastal urban planning after 2010 have been focused at the local level on anti-tsunami engineering solutions, whereas other policies like key infrastructures restriction in tsunami flood zones or relocation of housing and key equipment were not adopted, due to the fact that reconstruction plans were non-binding master plans and citizens scarcely participated in their development.

The research by Rogers et al. (2019) deals with coastal communities and describe NASA’s efforts on applying remote sensing and modelling in areas exposed to high potential risks. In particular, the NASA Disaster Program held a workshop in Spring 2017 with participants from academic institutions, local and regional which gave birth to the Mid-Atlantic Community and Area at Intensive Risk (CAIR) team that demonstrated the ability to integrate satellite derived earth observations and physical models into trusted and actionable knowledge for tactical and strategic decision-making.

From a different threat, Cradock-Henry et al. (2018) analyse the emergence of new networks, agents, and institutions after the 2016 earthquake, for the small coastal settlement of Kaikōura in New Zealand. The paper utilises a qualitative method based on 12 semi-structured interviews and two focus groups, supported by literature review and document analysis. The first interviewees were selected among existing research collaborations with emergency management staff and local council. Then, a snow-ball strategy was implemented to identify further participants’ representative of the range of affected interests, making this a convenient sample (e.g. local government, regional economic development staff, food producers). Themes were identified and emerged through the process of data analysis using deductive and inductive methods, validated by inter-reliability analysis. The paper focuses on two case studies that show the transformation of the rural community after the earthquake. In the first one, the shock induced by this event raised the awareness of the rural community of how important it was to strengthen local food networks, to build local capabilities, to promote local agricultural products as one of the reasons for tourism, the major economic driver of the district. The second case study regards the activity of a team that was formed after the earthquake to provide isolated rural households with life essentials and a readily accessible line of communication, responding to immediate personal needs.

On the importance of CIs for local communities, Grosse (2019) focuses on regional airports in Sweden, clarifying their role for communities, in terms of regional development and civil protection. Four semi-structured interviews, employing a questionnaire with open-ended questions, were conducted, and recorded among a selection of stakeholders. The data collection was extended with a workshop of 14 delegates from public and private organisations, which aimed at giving suggestions to develop a conceptual model for assessing risks, the criticality of infrastructure in the context of civil protection and the economic value of airports. In this case, the participants emphasised the importance of several transports by air for the society: ambulance flights, transportation of criminals, flights for crisis management such as fighting wildfires.

Another paper deals with the economic impacts on a community: Akhtar and Santos (2013) investigate the adverse impacts of hurricanes to interdependent workforce sectors in Virginia, using the dynamic inoperability input–output model (DIIM), a risk-based transformation of the Leontief’s classic input–output model (i.e. a system of linear equations). To study the ripple effects which lead to serious economic repercussions and to identify the most critical workforce sectors and prioritise them, two significant metrics were considered: inoperability level and economic loss to industry sectors. Although this research is based on quantitative method, qualitative sources, as published surveys of workforce absenteeism in the aftermath of hurricanes, constituted the core of the data analysis, being the source for the formulation of the workforce perturbation models. The results provide guidance in disaster policy-making, particularly in systems-based resource allocation, enhancing preparedness to better manage the consequences of hurricanes to workforce sectors.

### Urban resilience

This paragraph is dedicated to the analysis of the papers where resilience is addressed explicitly as referred to cities and urban development. On this context, resilience has a twofold interpretation: an umbrella term for everything to be addressed within a city, related to the aspects of sustainability, climate change adaptation, or disaster risk reduction, an ability required within a stable risk assessment process (Ferreira and Bellini 2018; Bellini et al. 2020). This second dimension is the one considering of interest in this review, since it is nested in the recovery phase after a hazard impact, representing the “bouncing back” process phase which is the literal translation of the term resilience that we discussed above (Fekete and Bogardi 2018).

On this research stream, both Fekete and Bogardi (2018) and Fekete and Fiedrich (2018) are chapters included in the book entitled: “Urban disaster resilience and security”. In the former, the authors underline the relevance of urban areas which are laboratories for observing and conceptualising resilience, because of the concentration and overlay of human beings, values, ideas with structural and non-structural objects, giving rise to socio-environmental or socio-ecological systems. In this environment, CIs have the potential to even aggravate a disaster situation for society, but are also key components for recovering, since they are a core part of resilience of an urban habitat. Again, Fekete and Fiedrich (2018) emphasise the central role of urban dimension where urban environment and resilient cities are flagships of recent research to investigate not only worst-case impacts of natural or man-made hazards but also to test the effectiveness of measures. Urban areas are selected for research and funding since density of people and human values are concentrated here and this is both an asset and risk factor. Finally, the authors show how existing indicators for resilience assessment can be improved or new indicators can be created: by adjusting old indicators, by involving experts or by using data sources or big data.

Gonzalez et al. (2017) report first-year findings of the European Union’s Horizon 2020 project called Smart Mature Resilience (SMR) (Iturriza et al. 2017; Marana et al. 2019). This project aims to develop a maturity model (a tool to assess current effectiveness of a group, supporting figuring out what needs to be improved) for society’s resilience, focusing on the progress towards resilience of seven cities, assimilated to the vertebrae of the strong European resilience backbone. In particular, the model consists of four maturity stages: Starting, Moderate, Robust, and verTebrate (SMART) corresponding to increasing cities’ capabilities towards higher resilience maturity levels. The model was enhanced through a literature survey on resilience, followed by an expert assessment by using Delphi method, and a series of workshops with experts on CIs, climate change, social dynamics (i.e. immigration, poverty, population ageing) and city representatives. Through Group Model Building, policies at strategic level were associated to each stage of the maturity model and classified along four resilience dimensions: Robustness of infrastructure & Resources, Preparedness, Leadership & Governance, Cooperation and Learning. The paper also describes an interactive questionnaire for risk assessment programmed in Excel (i.e. Systemic Risk Assessment Questionnaire), whose questions are dependent each other, capturing the interdependence between risks, and whose output is a risk score which helps in assessing a particular project or initiative and in prioritising those areas which require more attention. Finally, by using a Group Explorer decision support system, different views regarding the risks associated to CIs, climate change and social problems, were collected during the workshops.

Monstadt and Schmidt (2019) shed light on the key role of the urban governance in the German approach to the CIs’ protection. The study is based on literature and documentary analyses, interviews with 48 experts and workshops with practitioners from local utility companies and crisis management teams. The qualitative research pointed out some critical issues for the urban governance level: the German federal system (the Lander delegate the key operational tasks to the municipalities), the European regulatory market reforms (many public utilities have been privatised and many networked infrastructures have been unbundled into disintegrated companies), and the budget reduction (the companies are more likely to accept temporary revenue losses through supply disruptions during crises than to invest in the prevention of such events). These issues result in a lack of coordination and cooperation among the main stakeholders; scarce information sharing and awareness of other infrastructure locations and their potential vulnerability; different vision about intervention priorities, mitigation strategies, staff training, and crisis exercises.

Pescaroli (2018) explores the awareness of cascading risk, the possible mitigation measures, and the current levels of training among stakeholders of the city of London, all intended as measures for increasing resilience in crisis management. The research started from a workshop whose participants came from emergency response organisations, public utilities, businesses, and academia. The workshop included questions with multiple items to be evaluated through a standard Likert scale. To record the bottom-up stakeholders’ perspective, each section of the survey also included open questions for the answerers, asking suggestions for mitigation measures, training strategies, general comments on the workshop. The answers were analysed with SPSS software, considering respondents’ experience, affiliation, and gender; possible correlations between answers within sections and across sections were also searched. The findings confirm that the current crisis management approach to cascading risk is inadequate. London’s stakeholders are aware and concerned about cascading events and interdependencies, but they recognise that these issues are not sufficiently incorporated in the current policies, practices, and emergency management at large.

From a terrorist point of view, urban environment is a perfect target, since cities can be seen as nodes where people, ideas, value streams, and information meet. On this context, Heino et al. (2019) aim at better understanding the operational environment when a CI becomes a target of a terrorist attack. Therefore, two scenarios, which reflected the model for comprehensive security defined in the Finnish Security Strategy for Society, were elaborated during a workshop: water contamination and electricity disruption. The workshop, organised at the Police University College in Tampere, was facilitated by a modified Open Space method. Unlike the original method, participants did not suggest topics for discussion, but they were invited to discuss about formation of situation awareness, competencies, and resources in crisis management, crisis communication, and development of a new tool in terms of continuity management. The sixteen participants represented the key actors who would be involved in the workshop scenarios in real life. In order to enhance system’s resilience, the findings reaffirm the importance of a multi-agency situational awareness, shared among key actors, as an essential element in decision-making and the need for a more networked defence to face threats as organised crime, hybrid action, and terrorism.

The study in Räikkönen et al. (2016) is based on the research conducted in the INTACT and HARMONISE projects, both co-funded by the European Union (Doyle et al. 2018). The first project addresses the resilience of CIs to the challenges posed by extreme weather events, while the second one presents resilience enhancement methods for large-scale urban built infrastructures. The aim of the paper is to establish a systematic approach for conducting risk assessment of urban CIs and for calculating and comparing benefits and costs of measures. The proposed approach is flexible, encompassing not only a rigorous quantitative assessment, but also allowing for a semi-quantitative or qualitative assessment. For example, for CI and system modelling, since the technical information on CI core functions and processes is specific for each CI and system, cooperation with experts, who have the knowledge and the data access, is needed. Moreover, for risk estimation and evaluation, there are three different types of calculations: qualitative, semi-quantitative, and quantitative. Some methods aim at general mapping and understanding of potential consequences and impacts, others are based on very detailed analysis in the form of indexing and strict quantitative modelling; a mixture of this approaches is used in some cases.

Finally, Lomba-Fernández et al. (2019) propose a guide to help cities to become more resilient by considering urban CIs as key elements to cope with Climate Change (CC)-related crises and maintain citizens’ welfare. The research consisted of two phases: the conceptualisation and the development phases. In the first phase, a literature review was carried out, analysing scientific and grey literature articles and reports. In the second phase, a co-creation approach was adopted, through focus group method, to elicit information from experts in two cities in the Basque country (Spain). In particular, two workshops in each city were organised with 30 multidisciplinary experts to identify resilience building policies for improving urban CIs’ resilience level and to carry out a detailed analysis for classifying the CIs against CC impacts and for studying the interrelationships among CIs. Then, two pilot tests, one in each city, were carried out to review the guide and use it in a real context. Moreover, additional interviews with the heads of the environmental departments of eight towns which had not participated in the development phase were carried out to provide relevant feedbacks. The main result of this research is a guide to help cities to analyse their current situation and understand challenges and opportunities supporting the development of resilience-strengthening strategies.

## Discussion

This section summarises and discusses the outcomes of current works on qualitative research for CIs’ resilience as emerged from full-text analysis of reviewed documents. The discussed approaches explore macro-themes that resilience research on CIs should take into account, mainly in case of Tier I (or Tier II) approaches. Nevertheless, many of these discussion points remain valid even for more quantitative investigations, as for Tier III approaches (Linkov et al. 2018).

### Qualitative and quantitative, or qualitative alone?

Adopting a qualitative rather than a quantitative approach to study complex issues is not an easy choice: both approaches have their pros and cons. As mentioned in the introduction, this is a debated topic, which also emerges from the reviewed papers.

The choice to use qualitative or quantitative approach should be driven by the needs and desires of the decision makers who usually prefer having numbers on which base their decisions. For some type of analyses, e.g. cost–benefit, it is expected to have quantitative assessment (Matsika et al. 2016), while for others, there is much more flexibility. About quantitative sources, often claimed to lack data, it has been proposed to elicit knowledge from press releases and media, which are however considered sub-optimal sources. Press releases are issued from corporate documents and tend to put organisations in good light by minimising failures, while media reports look for the emotional and interesting issues of stories (Simpson et al. 2010). This means it is a viable option, but there should be a conscious usage of the respective storytelling. One possible option here, at least for some type of disruption and events, could be to link the usage of social media (Verma et al. 2019) (e.g. Twitter), possibly as a social sensor and get information on this direction. Some early results on power grids prove the potential benefits to further research on the topic (LaLone et al. 2017; Heglund et al. 2020).

Quantitative data offer an inherent benchmarking dimension, which anyway requires to be complemented by qualitative assessment to ask the right questions for understanding the context, and provide meaningful answers to interpret the scores (Bernroider et al. 2016). However, qualitative assessment needs also an attentive and accurate planning to avoid unmanageable results. In retrospect, it is worth mentioning how Bernroider et al. (2016) admit that questionnaire and terminology might have been more precise in their study if workshops and interviews should have been conducted first. Then, the original questionnaire should have been adjusted considering the respondents’ comments, allowing for a more precise questionnaire to be distributed. There is always a trade-off to understand the right sample size for preliminary interviews and workshops, which could count on the so-called knowledge saturation principle (Onwuegbuzie et al. 2011). Future research may thus investigate this principle explicitly in the context of CIs, to prove its feasibility and define guidelines to support analysts in conducting their research.

Some scepticism has been documented about resilience measurability in quantitative terms, because of CIs’ complexity which cannot be covered simply by larger data availability (Fekete and Fiedrich 2018). The same authors warn about the shortcomings of exemplary social science qualitative assessments, backed from their experience on the field. Using participative social science methods such as workshops, focus group discussions, and expert interviews, Fekete and Fiedrich (2018) realise that the same experts might offer contradicting arguments in assessments repeated only few weeks after the first one.

Reliability of quantitative scoring must be treated as cautiously as reliability upon individual qualitative results. Nevertheless, combining the two dimensions appears a promising choice to build bridges between users, social and natural scientists. Such mixed methods and approaches are often balancing demands by different end users, and even if they could disappoint some of them partly, they generally allow for a bigger picture.

### Involvement of stakeholders

Another relevant point of discussion is the involvement of all stakeholders, in both urban realities, and complex infrastructure systems, and organisational settings, characterised by dense interdependencies and hybridity between social, natural, and technological worlds (Monstadt and Schmidt 2019).

For example, the importance of preparedness and contingency thinking emerges explicitly in urban settings, along with the need for a networked defence strategy among stakeholders to face threats which lurk within the increased diverse and sophisticate operating environment (e.g. organised crime, hybrid action, and terrorism) (Heino et al. 2019). Resilience requires different strategies for urban realities (Dierich et al. 2019): while bigger cities usually have centralised resources to face crises, smaller ones depend even more strictly on the collective use of resources and, therefore, emphasise cooperation and coordination, including citizens’ participation (Lomba-Fernández et al. 2019). Additional examples on the need for stakeholders’ involvement refer to community resilience, where methods such as the Resilience Matrix (Fox-Lent et al. 2015; Linkov et al. 2018) or its variant Population Resilience Matrix (Rand et al. 2020) have been used to organise community goals in a context of population displacement and infrastructure reconstruction. About the Population Resilience Matrix, the authors explicitly claim as critical the community engagement for establishing legitimacy around acceptable bounds of performance.

A similar need for stakeholder involvement has been documented at intra- and inter-organisational levels to support stakeholders’ common knowledge and resilience to cascading failures (Seppänen et al. 2018). A noteworthy result in this case is the benefits of cooperation between administrative authorities, healthcare providers, and power suppliers to plan for the negative impacts of a disruption (Hiete et al. 2011). At the extremes of this concept there lies the push for mitigation measures to be included within legislation (Pescaroli 2018), and the involvement at local level, where private citizens may voluntarily self-arrange in groups to define some resilience strategy (Bouchon et al. 2014).

However, such type of coordination is a rather complicate and delicate task, and should be treated taking care of the potential privatisation of some companies, as well as the organisational cultures. A critical reflection in this sense comes from the STYREL project, where the public–private cooperation envisaged in the process revealed lack of trust between actors, concerning both lack of resources and feedbacks (Olausson 2019). Most reviewed papers shed light on the importance of data availability and information sharing. Few studies focus on extracting the failure dependencies on experts’ knowledge because detailed information about the CI failure interdependencies is considered highly sensitive and private CI operators are reluctant to share information with academic communities (Seppänen et al. 2018; Beheshtian et al. 2017).

Involvement means cooperation, information sharing, transparency, and discussion to enrich one other perspective. Communication is crucial at all level of CIs’ management (Antunes et al. 2017). Future research should explore the contributing success factors to cooperation as well as it should give evidence of those scenarios where cooperation drove to positive outcomes, isolating the strategies that foster success, and the ones that may create detrimental effects. A promising research stream in this sense may refer to the usage of methods built in the context of Resilience Engineering used for socio-technical system safety, as for the Functional Resonance Analysis Method, early applied for CI resilience in the urban context (Bellini et al. 2016). At societal, or even organisational level, this may imply the push for development of dedicated app to inform about vulnerabilities and response strategies, as for the early results in the disaster management domain (Petersen et al. 2019a).

### Guidelines, but for whom?

Several papers reveal the need of guidelines and criteria for helping authorities at different scales to develop crisis management, to analyse vulnerabilities, and to improve CIs’ resilience (Herrera et al. 2017; Woltjer et al. 2018). Of course, the societal scale at which guidelines are proposed shapes the details and operating strategies suggested (Petersen et al. 2020c).

At international scale, focusing on transportation infrastructure, it is worth mentioning how the European Surface Transport Operator (EUSTO) built common guidelines for developing security plans for surface transport with an EU dimension, involving National Contact Points of EU members and surface transport stakeholders (Hedel et al. 2018). Still in EU, the AESOP (Association of European Schools of Planning) guidelines offer details on identifying target population, and emphasise the need for CI operators to give evidence via news media of their positive working relationships with their counterparts, always in light of the regulatory context (Reilly et al. 2018).

At national scale, CIs require more regulatory efforts by national policies and highlight the need for identifying place-based vulnerabilities, locally differentiated preparedness strategies, and the training of local utility companies as well as crisis management teams (Monstadt and Schmidt 2019). Besides, it should also be fostered some public–private partnerships, leadership, trusted and secured information sharing, and exercises for social vulnerability assessment (Carpenter 2014).

At regional scale, guidelines should support authorities to integrate CI protection and reconstruction into the spatial planning process. This target can be achieved via indicators (e.g. population equivalents) for a systematic analysis of vulnerabilities, interdependencies, and criticalities of infrastructures and customers (Riegel 2014). This is a viable research stream, as proved by the interventions defined at a local level to increase community resilience in light of a multi-level integrated management approach, particularly careful to respect and empower local cultures and capabilities (Glavovic 2008) or by modelling population displacement as a function of infrastructure reconstruction decisions, in order to implement best strategies for infrastructure recovery (Rand and Fleming 2019). The local level also includes the management of urban resilience and the treats a stakeholder profile should respect to be involved in a resilience research (Lomba-Fernández et al. 2019).

In summary, the reviewed papers show the need of guidelines, and the benefits arising from them at different scales (Save et al. 2019), with no specific inter-level research available yet. Future research should thus start from these results and propose different staging areas to harmonise the priorities, and the constraints imposed by different competent authorities acting at different scales (international, nation, regional, local).

### Continuous trusted learning

A continuous trusted learning process is crucial for both organisations and communities. For these settings, it is frequently reported a poor risk awareness, as well as the absence of systematic knowledge sharing, and poor scenario training and emergency preparedness (Johnsen et al. 2009).

Solutions about organisations available in the literature refer to the preparation of inter-teams’ meetings, where diverse personnel are involved (technicians, operators, managers, etc.) to reinforce mutual understanding and foster participation, by complementing top-down decision-making with bottom-up evidences. It is expected that the ownership of solutions supports a learning-oriented healthy environment (Pathirage and Al-Khaili 2016). Similar evidence emerges in school emergency management. School staff do not know fully the actions and the post-evacuation procedures, and both staff and students do not share the correct perception of natural hazards. At a management level, this result calls for a reconsideration of the connection between schools’ evacuation plans and cities’ civil protection plans (Bandecchi et al. 2019). Risk awareness and learning-oriented approach are essential for communities who face natural hazards continuously, and have to deal with improvised means (Mavhura 2017; Cradock-Henry et al. 2018). As an example, a research on the Appalachian community proved their resilience based on faith and spirituality, cultural values and heritage, and social support despite stressors like poverty, rural isolation, low educational level (Banks et al. 2016). Similar results have been achieved by this type of research conducted in Zimbabwe where the results pinpoint to lemmas used by the community to identify inherent capacities and strategies (Mavhura 2017).

These observations show how helpful might be a precise type of qualitative research, designed for ethnographic research to understand local absorptive and adaptive capacities. Understanding humans in their real-life environment becomes a central point to incorporate anthropocentric assessment and transfer them to other communities. Besides validating the idea in many communities, future research may extend the concept to organisational ethnography (Yanow 2012).

### From cybersecurity to cyber resilience

Except for some papers like Linkov et al. (2013) where the Risk Matrix is adopted to develop metrics useful for assessing resilience of cyber systems, as explored from the bibliometric findings in Sect. 3, cybersecurity has been less investigated than others via qualitative research methods. Literature review shows that there is a focus on identifying cyber vulnerabilities and preventing cyber-attacks, but much less attention to mitigate their effects by improving cyber resilience. For example, Lykou et al. (2019) discovered that the majority of the available questions, among distributed qualitative surveys, focus on protection measures and technical safeguards rather than examining response and recovery strategies. Available Self-Assessment tools are only one—limited—component to assess cybersecurity: they should be revised to ensure the full resilience capabilities and customised based on the organisation profile (Lykou et al. 2019). Cybersecurity requires an evolution of traditional risk management process, calling for a greater emphasis on shared responsibility, leadership, and humans intended as resources (Rajamaki et al. 2018). The same observations apply for cyber resilience in space missions (Millwood 2019).

These early observations mean that cyber resilience among modern CIs is an under-developed domain, which requires the modern view on resilience inspired by other more traditional CIs. Qualitative research here plays a central role to prioritise activities and identify threats by means of dedicate surveys that can increase the level and quality of information security to next maturity levels.

## Conclusions

Continuing failures and disasters remind us the need to further advance the available scientific understanding, and policy-making, of resilience for CIs. The management of resilience in modern CIs requires an understanding of CIs’ functioning, as well as the needs and the determinant features of all the stakeholders involved. Modern CIs require indeed methodologies able to capture diversity, heterogeneity, and inter-relatedness, providing meaningful and interpretable representations also in light of cyber-physical systems’ interdependencies (Patriarca et al. 2021). Qualitative research has a high potential in such settings, as shown by the results of the systematic review presented in this paper. Through qualitative research, the analysis of CIs can reflect more explicitly the complex and tight coupled factors of individual, group, societal behaviours, as they act jointly with technological artefacts.

While the empirical results discussed in the paper prove the usability and usefulness of qualitative research to deal with resilience management of CIs, there are still several research areas to be developed, as linked to the notion of knoweldge integration: (i) from a methodological perspective, integration with quantitative methods still adopting a systemic perspective; (ii) from a management perspective, integration across different micro-meso-macro scales; (iii) from a strategical perspective, integration among data from different stakeholders, ensuring trust and cooperation; (iv) from a tactical perspective, integration and dissemination of knowledge to support a continuous trusted learning.

In our modern, uncertain, and turbulent world, future research on CIs’ resilience should prioritise integration to support the survivability and development of future organisations, communities, cities, and societies towards next staging areas of evolution and adaptations.

## Appendix 1

See Table 2

**Table 2:** Summary of reviewed papers

Authors	Title	Year	Main dimension	Domain	Approach
Banks L.H. Davenport L.A., Hayes M.H., McArthur M.A., Toro S.N., King C.E., Vazirani H.M	Disaster Impact on Impoverished Area of US: An Inter-Professional Mixed Method Study	2016	Community	Community	Interview; questionnaire; Ethnographic research
Glavovic B.C	Sustainable coastal communities in the age of coastal storms: Reconceptualising coastal planning as ‘new’ naval architecture	2008	Community	Not specified	Interview
Mavhura E	Applying a systems-thinking approach to community resilience analysis using rural livelihoods: The case of Muzarabani district, Zimbabwe	2017	Community	Community	Interview; questionnaire; Focus group; Observation
Ong J.M., Jamero M.L., Esteban M., Honda R., Onuki M	Challenges in Build-Back-Better Housing Reconstruction Programs for Coastal Disaster Management: Case of Tacloban City, Philippines	2016	Community	Community	Questionnaire; focus group; interview
Herrmann Lunecke M.G	Urban planning and tsunami impact mitigation in Chile after February 27, 2010	2015	Community	Urban infrastructures	Interview; questionnaire
Simpson D.M., Lasley C.B., Rockaway T.D., Weigel T.A	Understanding critical infrastructure failure: Examining the experience of Biloxi and Gulfport, Mississippi after Hurricane Katrina	2010	Community	Community	Interview
Rogers L., Borges D., Murray J., Molthan A., Bell J., Allen T., Bekaert D., Loftis J.D., Wang H., Cohen S., Sun D., Moore W	NASA’s Mid-Atlantic Communities and Areas at Intensive Risk Demonstration:: Translating Compounding Hazards to Societal Risk	2019	Community	Community	Workshop
Johnsen S.O., Øren A	10 years from risk assessment to regulatory action—is complacency creating a reactive and brittle regulatory regime in Norway?	2015	Community	Not specified	Interview
Grosse C	Airports as Critical Infrastructure: The Role of the transportation-by-air System for Regional Development and Crisis Management	2019	Community	Transportation systems; aviation	Interview; questionnaire; Workshop
Verma R., Karimi S., Lee D., Gnawali O., Shakery A	Newswire versus Social Media for Disaster Response and Recovery	2019	Community	Community	Social network
Murphy S.A., Brown J., Shankar A., Lichtveld M	A quantitative assessment of institutions of higher education disaster preparedness and resilience	2019	Community	Community	Questionnaire
Petersen L., Sjöström J., Horvath E	Evaluating critical infrastructure resilience via tolerance triangles: Hungarian Highway pilot case study	2019	Community	Transportation systems	Questionnaire
Bunney S.L., Ward S., Butler D	Can UK water service providers manage risk and resilience as part of a multi-agency approach?	2016	Community	Water facilities	Interview
Cradock-Henry N.A., Fountain J., Buelow F	Transformations for resilient rural futures: The case of Kaikōura, Aotearoa-New Zealand	2018	Community	Community	Interview; focus group
Akhtar R., Santos J.R	Risk-based input–output analysis of hurricane impacts on interdependent regional workforce systems	2013	Community	Not specified	Questionnaire
Pagán-Trinidad I., Lopez R.R., Diaz E.L	Education and building capacity for improving resilience of coastal infrastructure	2019	Community	Community	Workshop
Bouchon S., Urquhart J., Gibson R., BaMaung D., Dimauro C., Trucco P	The role of public–private stakeholder collaboration to achieve critical infrastructures resilience: Main findings from the EU-CIPS MIRACLE project	2014	Community	Not specified	Questionnaire; focus group; interview
Petersen L., Lange D., Theocharidou M	Who cares what it means? Practical reasons for using the word resilience with critical infrastructure operators	2020	Community	Not specified	Workshop; interview;
Rosenqvist H., Reitan N.K., Petersen L. Lange D	ISRA: IMPROVER societal resilience analysis for critical infrastructure	2018	Community	Logistics	Focus group; questionnaire
Adini B. et al	Striving to be resilient: What concepts, approaches and practices should be incorporated in resilience management guidelines	2017	Organisational	Aviation; healthcare	Delphi method; questionnaire
Van Laere J., Berggren P., Gustavsson P., Ibrahim O., Johansson B., Larsson A., Lindqwister T., Olsson L., Wiberg C	Challenges for critical infrastructure resilience: Cascading effects of payment system disruptions	2017	Organisational	Payment system	Interview; workshop
Labaka L., Comes T., Hernantes J., Sarriegi J.M., Gonzalez J.J	Implementation methodology of the resilience framework	2014	Organisational	Not specified	Interview; workshop; Delphi method; questionnaire
Pathirage C., Al-Khaili K	Disaster vulnerability of Emirati energy sector and barriers to enhance resilience	2016	Organisational	Energy systems; supply chain management; oil and gas infrastructures	Questionnaire; interview
Johansson B.J.E., Jaber A., Van Laere J., Berggren P	The lack of preparedness for payment disruptions in local community core businesses	2018	Organisational	Payment system	Interview
Mendonça D., Wallace W.A	Factors underlying organisational resilience: The case of electric power restoration in New York City after 11 September 2001	2015	Organisational	Energy systems	Interview; questionnaire
Brown C., Seville E., Vargo J	Measuring the organisational resilience of critical infrastructure providers: A New Zealand case study	2017	Organisational	Energy systems; water facilities; transportation systems; telecommunication networks; oil and gas infrastructures	Questionnaire
Field C., Look R	A value-based approach to infrastructure resilience	2018	Organisational	Not specified	Interview
Kachali H., Stevenson J.R., Whitman Z., Seville E., Vargo J., Wilson T	Organisational Resilience and Recovery for Canterbury Organisations after the 4 September 2010 Earthquake	2012	Organisational	Logistics; construction; IT networks	Questionnaire
Burbidge R	Adapting European airports to a Changing Climate	2016	Organisational	Transportation systems; aviation	Questionnaire; workshop
Hiete M., Merz M., Schultmann F	Scenario-based impact analysis of a power outage on healthcare facilities in Germany	2011	Organisational	Healthcare	Questionnaire
Comes T., Bertsch V., French S	Designing dynamic stress tests for improved critical infrastructure resilience	2013	Organisational	Energy systems	Questionnaire
Bandecchi A.E., Pazzi V., Morelli S., Valori L., Casagli N	Geo-hydrological and seismic risk awareness at school: Emergency preparedness and risk perception evaluation	2019	Organisational	Community	Questionnaire
Große C	Sources of uncertainty in Swedish emergency response planning	2019	Organisational	Energy systems	Interview; questionnaire
Petersen L., Fallou L., Carreira E., Utkin A	Public tolerance levels of transportation resilience: A focus on the Oresund region within the IMPROVER project	2018	Organisational	Transportation systems	Questionnaire
Sapeciay Z., Wilkinson S., Costello S.B., Adnan H	Building Organisational Resilience for the Construction Industy: Strategic Resilience Indicators	2019	Organisational	Construction	Questionnaire; interview
Förster P., Schachtebeck P.M., Feuerle T., Hecker P., Branlat M., Herera I., Woltjer R	An Approach for Attribute- and Performance-Based Evaluation of Interdependent Critical Infrastructures	2019	Organisational	Aviation; healthcare	Simulation game; focus group
Carpenter A.M	Critical infrastructure resilience: A baseline study for Georgia	2014	Organisational	Supply chain management; transportation systems; emergency response; water facilities; healthcare; energy systems; defence	Interview
Line M.B	A study of resilience within information security in the power industry	2013	Organisational	Energy systems	Interview
Kachali H., Seville E., Vargo J	Recovery and resilience of industry and geographic sectors after the 2010 and 2011 Canterbury earthquakes	2012	Organisational	Community	Interview
Olausson P.M	Planning for resilience in the case of power shortage: The Swedish STYREL policy	2019	Organisational	Energy systems	Questionnaire; interview
Räikkönen M., Molarius R., Mäki K., Forssén K., Petiet P., Nieuwenhuijs A	Creating stakeholder value through risk mitigation measures in the context of disaster management	2017	Organisational	Energy systems	AHP
Berggren P., Van Laere J., Johansson B.J	Using a mixed-methods assessment approach in a gaming-simulation environment to increase resilience	2018	Organisational	Payment system	Simulation game; questionnaire
Linkov I., Palma-Oliveira J.M	An Introduction to resilience for critical infrastructures	2017	Organisational	Not specified	Workshop
Cedrini V., Mancini M., Rosi L., Mandarino G., Giorgi S., Herrera I., Branlat M., Pettersson J., Jonson C.-O., Save L., Ruscio D	Improving resilience management for critical infrastructures—strategies and practices across air traffic management and healthcare	2018	Organisational	Aviation; healthcare	Delphi method
Pathirage C	The role of knowledge management in effective disaster mitigation strategies: Critical infrastructure	2010	Organisational	Not specified	Qualitative analysis
Save L., Branlat M., Hynes W., Bellini E., Ferreira P., Lauteritz J.P., Gonzalez J.J	The Development of Resilience Management Guidelines to Protect Critical Infrastructures in Europe	2019	Organisational	Aviation; healthcare	Workshop; interview; questionnaire; simulation game
Woltjer R., Hermelin J., Nilsson S., Oskarsson P.-A., Hallberg N	Using requirements engineering in the development of resilience guidelines for critical infrastructure	2018	Organisational	aviation; healthcare	Delphi method; interview; workshop; focus group
Herrera I., Grøtan T.O., Woltjer R., Nevhage B., Nilsson S., Trnka J., Adini B., Cohen O., Forsberg R., Jonson C.O	Applying resilience concepts in crisis management and critical infrastructures—The DARWIN project	2017	Organisational	Aviation; healthcare	Interview
Petersen L., Lundin E., Fallou L., Sjöström J., Lange D., Teixeira R., Bonavita A	Resilience for whom? The general public’s tolerance levels as CI resilience criteria	2020	Organisational	Water facilities	Questionnaire; interview
Storesund K., Reitan N.K., Sjöström J., Rød B., Guay F., Almeida R., Theocharidou M	Novel methodologies for analysing critical infrastructure resilience	2018	Organisational	Water facilities	Focus group; interview; questionnaire
Lange D., Honfi D	Novel techniques and approaches for risk based application of resilience concepts to critical infrastructure: An introduction to the IMPROVER project	2017	Organisational	Community	Questionnaire
Hsieh C.-H., Tai H.-H., Lee Y.-N	Port vulnerability assessment from the perspective of critical infrastructure interdependency	2014	Techno-centric	Transportation systems	Interview; questionnaire; workshop; AHP; Delphi method
Cutts M., Wang Y., Yu Q	New Perspectives on Building Resilience into Infrastructure Systems	2017	Techno-centric	Transportation systems; energy systems; supply chain management; water facilities; telecommunication networks	Workshop; focus group
Münzberg T., Wiens M., Schultmann F	A spatial–temporal vulnerability assessment to support the building of community resilience against power outage impacts	2017	Techno-centric	Energy systems	Delphi method; workshop; questionnaire
Petit F.D., Eaton L.K., Fisher R.E., McAraw S.F., Collins III M.J	Developing an index to assess the resilience of critical infrastructure	2012	Techno-centric	Not specified	Questionnaire
Feldpausch-Parker A.M., Peterson T.R., Stephens J.C., Wilson E.J	Smart grid electricity system planning and climate disruptions: A review of climate and energy discourse post-Superstorm Sandy	2018	Techno-centric	Energy systems	Focus group
Münzberg T., Wiens M., Schultmann F	The effect of coping capacity depletion on critical infrastructure resilience	2015	Techno-centric	Energy systems	Questionnaire
Bloomfield R.E., Popov P., Salako K., Stankovic V., Wright D	Preliminary interdependency analysis: An approach to support critical-infrastructure risk-assessment	2017	Techno-centric	Energy systems; telecommunication networks	Questionnaire
Sircar I., Sage D., Goodier C., Fussey P., Dainty A	Constructing Resilient Futures: Integrating UK multi-stakeholder transport and energy resilience for 2050	2013	Techno-centric	Transportation systems; energy systems; supply chain management	Interview; focus group
Johnsen S., Skramstad T., Hagen J	Enhancing the safety, security and resilience of ICT and SCADA systems using action research	2009	Techno-centric	Supply chain management	Questionnaire
Valiquette L'Heureux A., Therrien M.-C	Interorganizational dynamics and characteristics of critical infrastructure networks: The study of three critical infrastructures in the greater Montreal area	2013	Techno-centric	Transportation systems; energy systems; telecommunication networks	Questionnaire
McBurnett L.R., Hinrichs M.M., Seager T.P., Clark S.S	Simulation Gaming Can Strengthen Experiential Education in Complex Infrastructure Systems	2018	Techno-centric	Water facilities	Workshop; simulation game; interview
Petersen L., Lundin E., Sjöström J., Lange D., Teixeira R	Creating comparable public tolerance and technical performance measures for critical infrastructure resilience evaluation	2018	Techno-centric	Water facilities	Interview; questionnaire
Bernroider E.W.N., Margiol S., Taudes A	Towards a general information security management assessment framework to compare cyber-security of critical infrastructure organizations	2016	Techno-centric	Cybersecurity	Workshop; interview; questionnaire
Leu P.O., Peter D	Case study: Information flow resilience of a retail company with regard to the electricity scenarios of the sicherheitsverbundsübung schweiz (Swiss security network exercise) SVU 2014	2016	Techno-centric	Supply chain management	Interview
Trucco P., Ward D	A clustering approach to the operational resilience analysis of Key Resource Supply Chains (KRSC): The case of Fast Moving Consumer Goods	2011	Techno-centric	Supply chain management	Interview; questionnaire
Lykou G., Anagnostopoulou A., Stergiopoulos G., Gritzalis D	Cybersecurity self-assessment tools: Evaluating the importance for securing industrial control systems in critical infrastructures	2019	Techno-centric	Cybersecurity; IT networks	Questionnaire
Landegren F., Höst M., Möller P	Simulation based assessment of resilience of two large-scale socio-technical IT networks	2018	Techno-centric	IT networks	Focus group; interview
Hedel R., Boustras G., Gkotsis I., Vasiliadou I., Rathke P	Assessment of the European programme for critical infrastructure protection in the surface transport sector	2018	Techno-centric	Transportation systems	Workshop; interview; questionnaire
Adelmeyer M., Teuteberg F	Cloud computing adoption in critical infrastructures—Status Quo and elements of a research agenda	2018	Techno-centric	IT networks	Interview
Reilly P., Serafinelli E., Stevenson R., Petersen L., Fallou L	Enhancing critical infrastructure resilience through information-sharing: Recommendations for European critical infrastructure operators	2018	Techno-centric	Not specified	Interview; focus group
Rajamaki J., Nevmerzhitskaya J., Virag C	Cybersecurity education and training in hospitals: Proactive resilience educational framework (Prosilience EF)	2018	Techno-centric	Healthcare; cybersecurity	Workshop
Seppänen H., Luokkala P., Zhang Z., Torkki P., Virrantaus K	Critical infrastructure vulnerability—A method for identifying the infrastructure service failure interdependencies	2018	Techno-centric	Energy systems; telecommunication networks	Workshop; questionnaire
Badea D., Bârsan G., Virca I., Iancu D	Quantitative and qualitative differences worth considering in approaching critical infrastructures resilience	2017	Techno-centric	Not specified	AHP
Student L.R., Goubran R., Kwamena F	Computer vision-assisted human-in-the-loop measurements: Augmenting qualitative by increasing quantitative analytics for CI situational awareness	2018	Techno-centric	energy systems; telecommunication networks	Social network
Vugrin E.D., Warren D.E., Ehlen M.A., Camphouse R.C	A framework for assessing the resilience of infrastructure and economic systems	2010	Techno-centric	Community	Qualitative analysis
Vugrin E.D., Warren D.E., Ehlen M.A	A resilience assessment framework for infrastructure and economic systems: Quantitative and qualitative resilience analysis of petrochemical supply chains to a hurricane	2011	Techno-centric	Supply chain management	Qualitative analysis
Comes T., Van De Walle B	Measuring disaster resilience: The impact of hurricane sandy on critical infrastructure systems	2014	Techno-centric	Transportation systems; energy systems; supply chain management	Qualitative analysis
Hughes L., de Jong M., Wang X.Q	A generic method for analyzing the risks to energy systems	2016	Techno-centric	Energy systems	Interview
Beheshtian A., Donaghy K.P., Geddes R.R., Rouhani O.M	Planning resilient motor-fuel supply chain	2017	Techno-centric	Supply chain management	Social network; interview
Matsika E., O'Neill C., Battista U., Khosravi M., Laporte A.D.S., Munoz E	Development of Risk Assessment Specifications for Analysing Terrorist Attacks Vulnerability on Metro and Light Rail Systems	2016	Techno-centric	Transportation systems	Workshop
Haass M.J., Warrender C.E., Burnham L., Jeffers R.F., Stevens-Adams S.M., Cole K.S., Forsythe C	Toward an Objective Measure of Automation for the Electric Grid	2015	Techno-centric	Energy systems	Interview
Riegel C	Infrastructure resilience through regional spatial planning—prospects of a new legal principle in Germany	2014	Techno-centric	Urban infrastructures	Qualitative analysis;
Gheorghe A.V., Georgescu A., Bucovețchi O., Lazăr M., Scarlat C	New Dimensions for a Challenging Security Environment: Growing Exposure to Critical Space Infrastructure Disruption Risk	2018	Techno-centric	Space systems	Qualitative analysis
Pfeiffer K.B., Burdi C., Schlueter S	Local supply chains: The disaster management perspective	2017	Techno-centric	Energy systems; water facilities; supply chain management	Questionnaire
Bellini E., Gaitanidou E., Bekiaris E., Ferreira P	The RESOLUTE project’s European Resilience Management Guidelines for Critical Infrastructure: development, operationalisation and testing for the urban transport system	2020	Urban	Transportation systems	Focus group
Ferreira P., Bellini E	Managing interdependencies in critical infrastructures—a cornerstone for system resilience	2018	Urban	Transportation systems	Interview
Pescaroli G	Perceptions of cascading risk and interconnected failures in emergency planning: Implications for operational resilience and policy making	2018	Urban	Not specified	Workshop; questionnaire;
Monstadt J., Schmidt M	Urban resilience in the making? The governance of critical infrastructures in German cities	2019	Urban	Urban infrastructures	Interview; workshop
Heino O., Takala A., Jukarainen P., Kalalahti J., Kekki T., Verho P	Critical infrastructures: The operational environment in cases of severe disruption	2019	Urban	Energy systems; water facilities	Workshop
Dierich A., Tzavella K., Setiadi N.J., Fekete A., Neisser F	Enhanced crisis-preparation of critical infrastructures through a participatory qualitative-quantitative interdependency analysis approach	2019	Urban	Energy systems; water facilities; emergency response	Interview; focus group
Gonzalez J.J., Bång M., Eden C., Gimenez R., Hernantes J., Howick S., Maraña P., Pyrko I., Radianti J., Rankin A., Sarriegi J.M	Stalking resilience: Cities as vertebrae in society’s resilience backbone	2017	Urban	Urban infrastructures	Workshop; Delphi method; questionnaire
Lomba-Fernández C., Hernantes J., Labaka L	Guide for climate-resilient cities: An urban critical infrastructures approach	2019	Urban	Urban infrastructures	Focus group; workshop; interview
Fekete A., Fiedrich F	Synthesis	2018	Urban	Urban infrastructures	Workshop; focus group; interview
Räikkönen M., Mäki K., Murtonen M., Forssén K., Tagg A., Petiet P.J., Nieuwenhuijs A.H., McCord M	A holistic approach for assessing impact of extreme weather on critical infrastructure	2016	Urban	Not specified	Qualitative analysis
Fekete A., Bogardi J.J	Considerations about urban disaster resilience and security—Two concepts in tandem?	2018	Urban	Urban infrastructures	Qualitative analysis
Marana P., Eden C., Eriksson H., Grimes C., Hernantes J., Howick S., Labaka L., Latinos V., Lindner R., Majchrzak T.A., Pyrko I., Radianti J., Rankin A., Sakurai M., Sarriegi J.M., Serrano N	Towards a resilience management guideline — Cities as a starting point for societal resilience	2019	Urban	Urban infrastructures	Questionnaire
Bellini E., Nesi P., Pantaleo G., Venturi A	Functional resonance analysis method based-decision support tool for urban transport system resilience management	2016	Urban	Transportation systems	AHP; interview

## Footnotes

1 A Likert scale is a psychometric scale commonly used to scaling responses in a survey research. When responding to a Likert item, respondents specify their level of agreement or disagreement on a symmetric agree–disagree scale for a series of statements. Thus, the range captures the intensity of their feelings for a given item.
